# Microspectrofluorimetry to dissect the permeation of ceftazidime in Gram-negative bacteria

**DOI:** 10.1038/s41598-017-00945-8

**Published:** 2017-04-20

**Authors:** Anas Allam, Laure Maigre, Julia Vergalli, Estelle Dumont, Bertrand Cinquin, Rodolphe Alves de Sousa, Jelena Pajovic, Elizabeth Pinet, Nikaia Smith, Jean-Philippe Herbeuval, Matthieu Réfrégiers, Isabelle Artaud, Jean-Marie Pagès

**Affiliations:** 1 UMR8601, LCBPT, CNRS Université Paris Descartes, Paris, France; 2 UMR_MD1, Aix Marseille Univ, IRBA, TMCD2 Facultés de Médecine et de Pharmacie, Marseille, France; 3 DISCO beamline, Synchrotron Soleil, Saint-Aubin, France; 4 Bertrand Cinquin, LBPA, ENS CACHAN, Cachan, France. Anas Allam, Pharma5, Casablanca, Morocco

## Abstract

A main challenge in chemotherapy is to determine the *in cellulo* parameters modulating the drug concentration required for therapeutic action. It is absolutely urgent to understand membrane permeation and intracellular concentration of antibiotics in clinical isolates: passing the membrane barrier to reach the threshold concentration inside the bacterial periplasm or cytoplasm is the pivotal step of antibacterial activity. Ceftazidime (CAZ) is a key molecule of the combination therapy for treating resistant bacteria. We designed and synthesized different fluorescent CAZ derivatives (CAZ*, CAZ**) to dissect the early step of translocation-accumulation across bacterial membrane. Their activities were determined on *E. coli* strains and on selected clinical isolates overexpressing ß-lactamases. The accumulation of CAZ* and CAZ** were determined by microspectrofluorimetry and epifluorimetry. The derivatives were properly translocated to the periplasmic space when we permeabilize the outer membrane barrier. The periplasmic location of CAZ** was related to a significant antibacterial activity and with the outer membrane permeability. This study demonstrated the correlation between periplasmic accumulation and antibiotic activity. We also validated the method for approaching ß-lactam permeation relative to membrane permeability and paved the way for an original matrix for determining “Structure Intracellular Accumulation Activity Relationship” for the development of new therapeutic candidates.

## Introduction

With the misuse and overuse of the various antibiotic families and the scarcity of new drugs available, multidrug resistance is now a major bacterial threat to healthcare worldwide^1–4^. A major concern discussed in various studies is the intracellular concentration of the antibiotic molecule^5–7^. In Gram-negative bacteria, the challenge for a drug is to permeate the outer membrane (OM) that protects the cell against external attacks^8, 9^. This membrane plays a key role in controlling the diffusion of external toxic molecules and the antibiotics must translocate through the outer membrane to reach the periplasmic target or additionally pass the inner membrane to reach the cytoplasmic target^10, 11^. During their translocation, the molecules can use various hydrophilic channels such as porins that form trimeric hydrophilic pores present in the OM^7, 8^.

Today a key challenge for hospital microbiologists is to bypass the resistance in a Gram-negative strain that can produce carbapenemase and/or ß-lactamase^12, 13^. Recent studies proposed an alternative way by using a combination of ceftazidime (CAZ) + ß-lactamase inhibitor^14–16^. Regarding this strategy, it is urgently required to dissect the relationships between the role of membrane and enzymatic barrier and the antibacterial activity of CAZ as recently reported^17^. Presently, various groups have studied the binding of ß-lactams to penicillin binding proteins (PBP) using fluorophore-conjugated-penicillin, termed Bocillin^18, 19^, fluorescent cephalosporin C^20^ or fluorescent meropenem^21^. Despite these various efforts to develop labeled antibiotic probes, no key information has been obtained regarding the role of porins, penetration rate, and the effect of ß-lactamase on periplasmic concentrations when addressing the choice of combination of clinically-used cephalosporin and ß-lactamase inhibitor in resistant bacterial cells. These concerns are of importance regarding the rate of ß-lactams penetration and accumulation close to their target and our objective is to develop a method allowing studying the early step of periplasmic accumulation of CAZ. Recently, Cinquin *et al*. have studied fluoroquinolone accumulation in the bacterial population and in single bacterial cells and demonstrated the impact of efflux transporters in the intra-bacterial accumulation^22^.

To study the periplasmic accumulation of CAZ, we adapted the method of single cell fluorescence imaging for fluoroquinolones^22^ to link the activity of CAZ to its permeation rate and periplasmic accumulation.

To this aim, we synthesized two fluorescent CAZ derivatives. Firstly, we labeled CAZ* by introducing a 7-dimethylaminocoumarin-4-acetic acid fluorophore *via* a glycine spacer to the 2-aminothiazol part of ceftazidime. We also prepared the corresponding sulfoxide CAZ*(S = O) and compared the activities of CAZ* and CAZ*(S = O) to unlabeled CAZ and CAZ(S = O) against *E. coli* strains (parental and AcrAB- derivative) and resistant clinical isolates. Then, we measured by microfluorimetry their uptake in these *E. coli* clinical isolates in the absence or in the presence of Polymyxin B Nonapeptide (PMBN), a membrane permeabilizer that has been previously used in combination with cephalosporins or other antibiotics^6, 23^.

In a second step, we designed CAZ** by introducing a 6-methoxyquinoline (6-MeOQ) fluorophore at the level of the leaving group, whose release is related to the β-lactam ring-opening by β-lactamase that is located in the periplasm, as probe of the correct localization and targeting. By using porin−/+ strains, membrane permeabilizer and ß-lactamase inhibitors, the kinetics and fluorescence measurements demonstrate that CAZ** is correctly addressed to periplasmic space and its PBP targets and this location fits in well with the antibacterial activity assayed under the same conditions.

This represents an original method to follow the translocation of cephalosporins from the extracellular medium to their periplasmic target and paves the way for innovative comparative studies to design the best chemical structure candidates improving periplasmic accumulation of antibacterial molecules.

## Results

Our objective is to generate a probe based on CAZ that allows monitoring the membrane translocation and the location in the periplasmic space. Two independent strategies have been selected: (i) CAZ* that corresponds to a labeled side chain remaining associated with the cephalosporin backbone but decreasing the activity, and, (ii) CAZ** for which the fluorescence signal is directly related to the ß-lactamase activity but, due to the small size of the generated dye, the fluorescence is observed in external medium.

### Probes design and fluorescence properties

**Figure 1:**
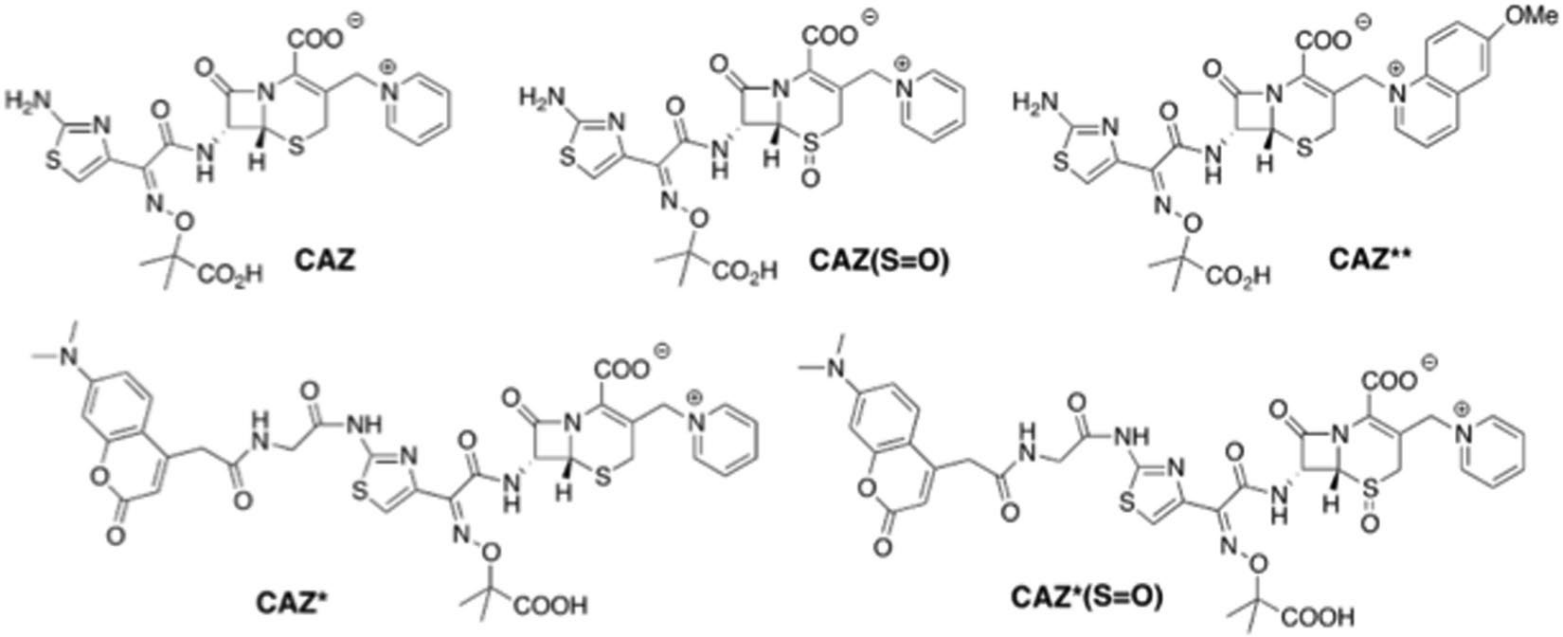
Structures of CAZ derivatives and fluorescent probes.

We first labeled CAZ by a coumarin probe grafted at the level of the amine of the aminothiazole through a glycine spacer (Fig. 1). The resulting CAZ* showed in fluorescence spectroscopy an emission spectrum with a maximum at 480 nm in phosphate buffer (pH 7.5) upon excitation either in deep UV at 270 nm or in near UV at 376 nm (Fig. 1S Supplementary Information). The only difference relies on the fluorescence intensity that is reduced by half when excited in deep UV relative to near UV. Two techniques to monitor the accumulation of CAZ* in bacteria were used, either epifluorescence microscopy with excitation light wavelengths from 360 to 400 nm or UV microfluorimetry that has a higher resolution upon selective excitation at 372 nm using the Synchrotron DISCO beamline facility available at SOLEIL (Gif sur Yvette, France).

To compare the activities of CAZ and CAZ* to those of their sulfoxides, we also prepared the corresponding sulfoxides CAZ(S = O) and CAZ*(S = O) (Fig. 1 and Scheme S1 in Supplementary Chemical Information). Cephalosporin sulfoxides are effectively reported to be active towards *Enterobacteriaceae* species, that are Gram-negative bacteria, and to be more stable *in cellulo* since they are less sensitive to β-lactamases than common cephalosporins^24^.

**Figure 2:**
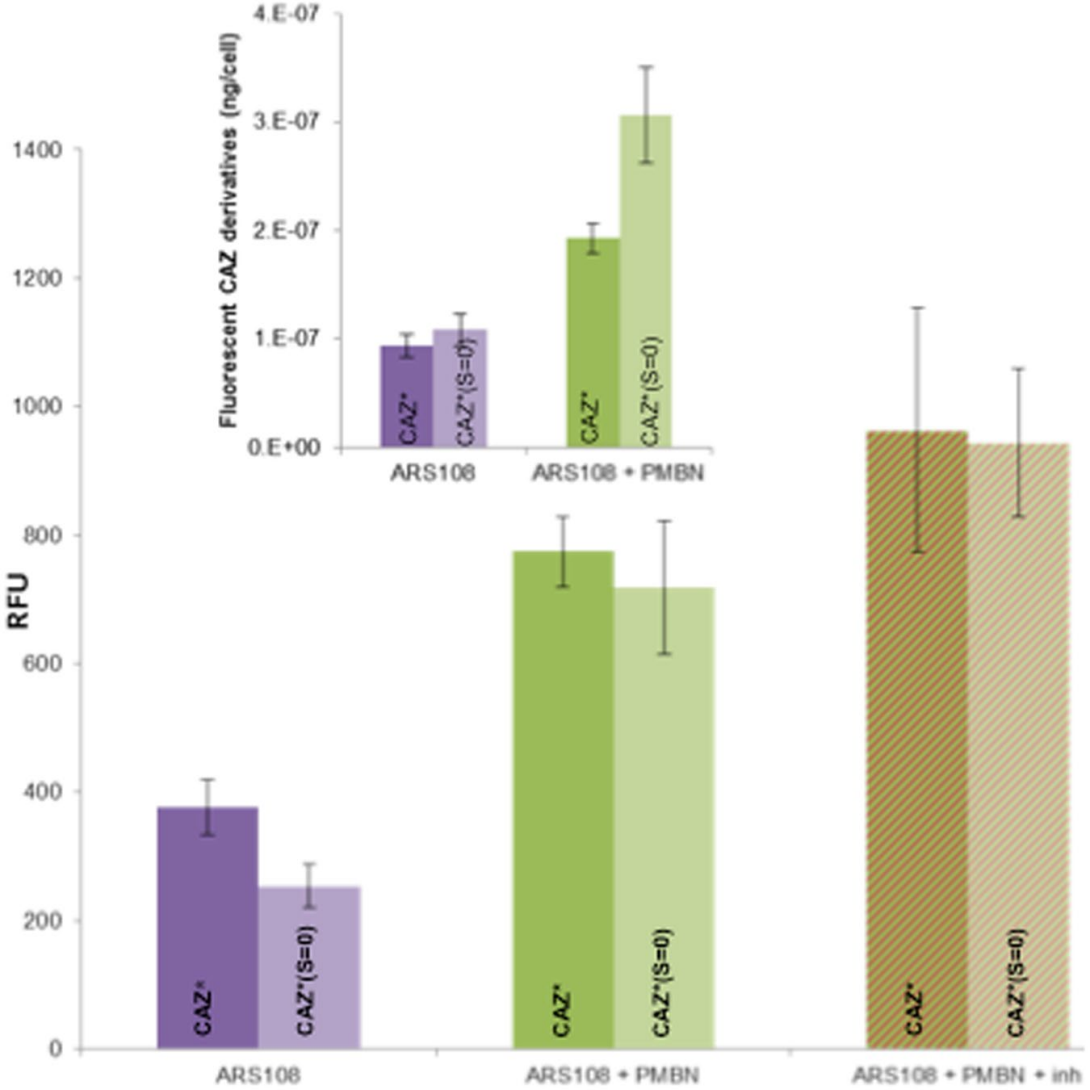
CAZ* and CAZ*(S = O) accumulation in bacterial population (measured on cell lysates after an incubation of 30 min). Normalized fluorescence signal corrected by the tryptophan signal and with negative controls (without CAZ* or CAZ*(S = O)). **Insert:** Intracellular concentration of CAZ* and CAZ*(S = O) obtained with normalization by the slope of their respective standard curve (Fig. 5S). CAZ* and CAZ*(S = O) concentrations: 32 mg/L. PMBN: final concentration 102.4 mg/L. Inh: tazobactam + clavulanic acid 4 mg/L each. Excitation wavelength 275 nm, emission peak measured at 450 nm. The columns with bars (standard deviations) corresponded to measurements carried out in triplicate.

As discussed below, introducing a bulky fluorophore on the aminothiazole moiety resulted in a partial loss of CAZ* antibacterial activity relative to the parent ceftazidime CAZ. In relevant crystal structures of PBP in complex with ceftazidime^25^, this amino group has proved to be essential for the interaction of ceftazidime with PBPs, by creating a strong hydrogen-bond network with several residues. To recover these specific interactions, we chose to introduce the fluorophore at the position of the cleavable group associated with the covalent binding of ceftazidime to PBPs or β-lactamase following the β-lactam ring opening. The pyridine moiety was replaced by the fluorescent 6-methoxyquinoline (6-MeOQ) (Fig. 1). Scheme S3 (Supplementary Chemical Information mation) outlined the synthetic pathways of this new cephalosporin derivative CAZ**. Upon excitation at 320 nm or 272 nm, 6-MeOQ and CAZ** are characterized in phosphate buffer (pH 7) by an emission at 370 nm, but with a much lower intensity for CAZ**. This allows us to specifically monitor the fluorescence of the side chain, 6-MeOQ, that is unmasked after the ß-lactamase cleavage occurring in the periplasm. Thus, the resulting signal illustrates the correct location of the drug in the subcellular compartment (Fig. 2S Supplementary Information).

### Antibacterial activities and porins

The activities of the various ceftazidime derivatives were assayed on the *E. coli* AG100 and AG100A (AcrAB- derivative) strains and the resistant clinical isolates ARS108 and ARS144 (Table 1 and Table 1S Supplementary Information). In general, the modified CAZ (CAZ* and CAZ*(S = O)) were less active on AG strains compare to intact CAZ. In contrast, a better activity was obtained with CAZ(S = O) alone on the resistant isolate. Moreover, the antibacterial activity of the tested molecules, CAZ and CAZ derivatives, was not significantly changed by the expression of the AcrAB pump in AG100 versus AG100A that is AcrAB- (Table 1).

**Table 1:** *E. coli* strains susceptibilities (in mg/L) to ceftazidime and modified-ceftazidimes.

Porin	AG100*	AG100A*	ARS108^#^	ARS144^#^
	+	+	−	+
AcrAB efflux pump	+	−	+	+
**ß-lactamase**	AmpC basal	AmpC basal	AmpC CTX-M-15	AmpC CTX-M-15 DHA-1
**CAZ**	0.5	0.25	64	1024
+PMBN	0.06	0.06	0.5	32
+Inh	0.25	0.25	2	4
+Inh + PMBN	0.06	0.06	0.03	0.25
CAZ*	64	16	>128	>128
+PMBN	8	4	64	128
+Inh	64	16	>128	16
+Inh + PMBN	4	4	4	8
CAZ**	2	1	64	>64
+PMBN	1	<0.125	4	>64
+Inh	1	0.5	4	8
+Inh + PMBN	<0.125	<0.125	0.125	1

*^,#^Isogenic strains [AG100 (parental) and AG100A, AcrAB- derivative] and clinical isolates^17, 23, 42^.

Porins and efflux components identified by Western Blot-immunodetection (OmpC or OmpF, AcrAB): -, no signal (*e.g*. no OmpC, no AcrAB).

CAZ, ceftazidime, CAZ* coumarin-ceftazidime, CAZ** 6-MeOQ-ceftazidime.

PMBN final concentration 51.2 mg/L for AG and 102.4 mg/L for ARS strains.

Inh: tazobactam + clavulanic acid, 4 mg/L each.

Values are medians of at least three independent experiments and are presented in mg/L.

Regarding the strain ARS108, in the presence of the double combination, inhibitors + permeabilizer, an additional gain of sensitivity was obtained for CAZ* when compared with permeabilizer alone. This was also the case with intact CAZ combined to inhibitors + permeabilizer (Table 1). Interestingly, a similar susceptibility was obtained with CAZ*(S = O) + PMBN on the two lab strains and the resistant isolate ARS108 (MIC = 8 mg/L) suggesting that the combination was able to circumvent the barrier due to porin absence. With CAZ(S = O) and CAZ*(S = O), the increased stability of sulfoxide CAZ towards β-lactamases can explain the lack of inhibitor effect on the susceptibility level (Table 1 and Table 1S Supplementary Information)^24^.

With CAZ**, it is interesting to mention that its activity on the susceptible strains is notably better than that obtained with CAZ* and the resulting MICs were close to those of CAZ (Table 1). Regarding the activity on resistant clinical isolate, the profile was also comparable to the CAZ ones especially in combination with permeabilizer and inhibitors. In clinical strains, the inhibitors significantly contribute to increase the bacterial susceptibility in addition to PMBN. This suggests that the only modification of the side chain in CAZ**/CAZ does not significantly modify the interactions with PBPs and so, the antibacterial activity. Consequently, this CAZ derivative can be used as a good reporter for both activity and periplasmic location.

### Outer membrane permeability, porin and nitrocefin hydrolysis

**Figure 3:**
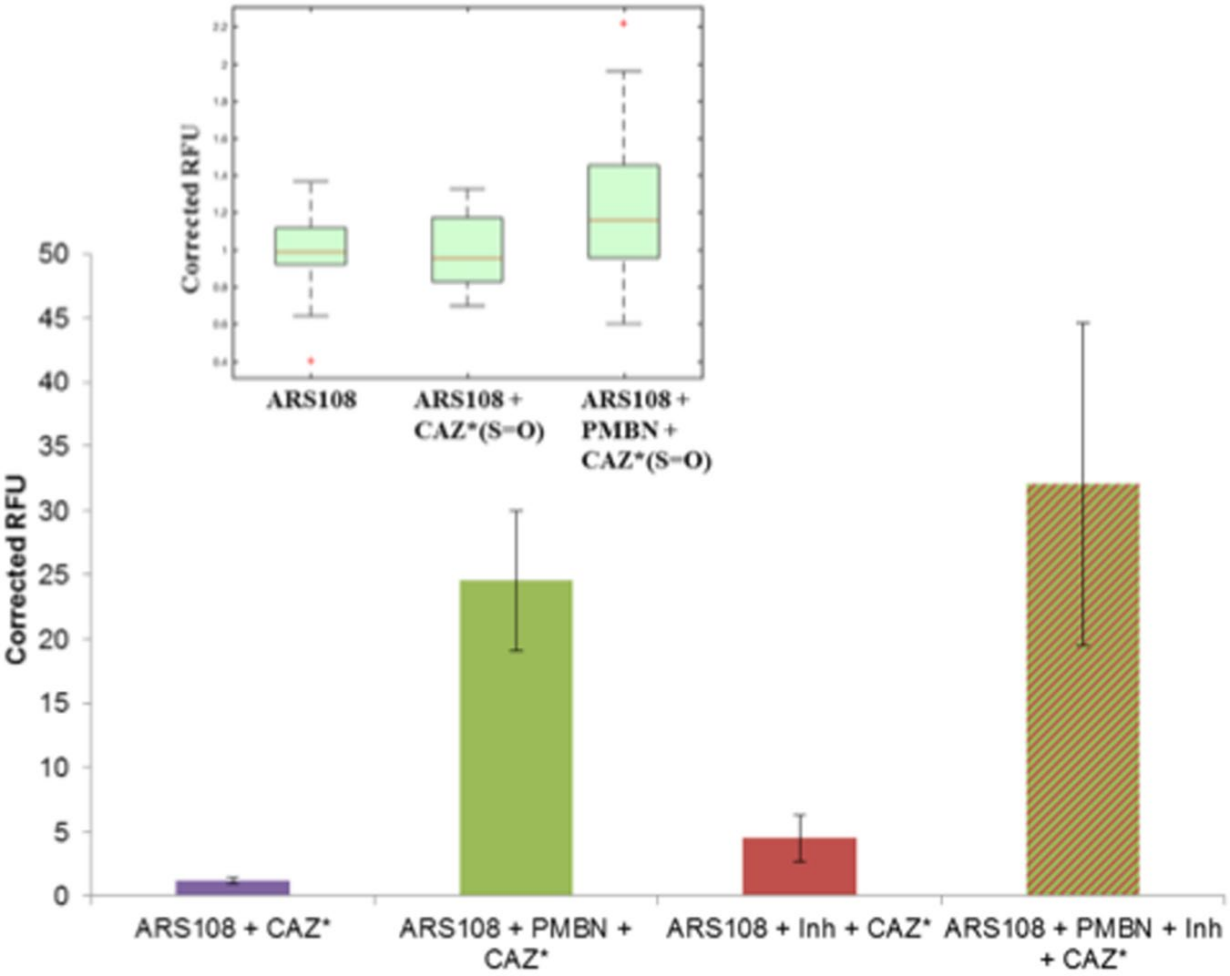
CAZ* and CAZ*(S = O) accumulation in individual bacterial cells (obtained after CAZ* and CAZ*(S = O) accumulation). Microspectrofluorimetry in individual bacteria (Excitation wavelength 372 nm, emission measured at 420–480 nm); corrected by the tryptophan signal and with negative controls (without CAZ*). CAZ* and CAZ*(S = O) concentrations: 32 mg/L. PMBN: final concentration 102.4 mg/L. Inh: tazobactam + clavulanic acid 4 mg/L each. The columns with bars (standard deviations) corresponded to measurements carried out with about 100 cells. **Insert:** Accumulation of CAZ*(S = O) corrected by the background and with negative controls (without CAZ*(S = O)). Boxplot corresponded to measurements carried out with about 30 cells.

In order to evaluate the involvement of porin during nitrocefin uptake, we compared two strains containing or not the OmpC/OmpF porins^17^. The kinetics of nitrocefin hydrolysis was recorded in the absence or in the presence of PMBN that increases CAZ susceptibility. Figure 3S (Supplementary Information) clearly showed that the PMBN speeded up the rate of nitrocefin hydrolysis in the porin- strain (ARS108) and in these conditions the curve was quite similar to that obtained in the porin + strain (ARS144). In the presence of inhibitor, the activity was drastically reduced in the two strains.

**Figure 4:**
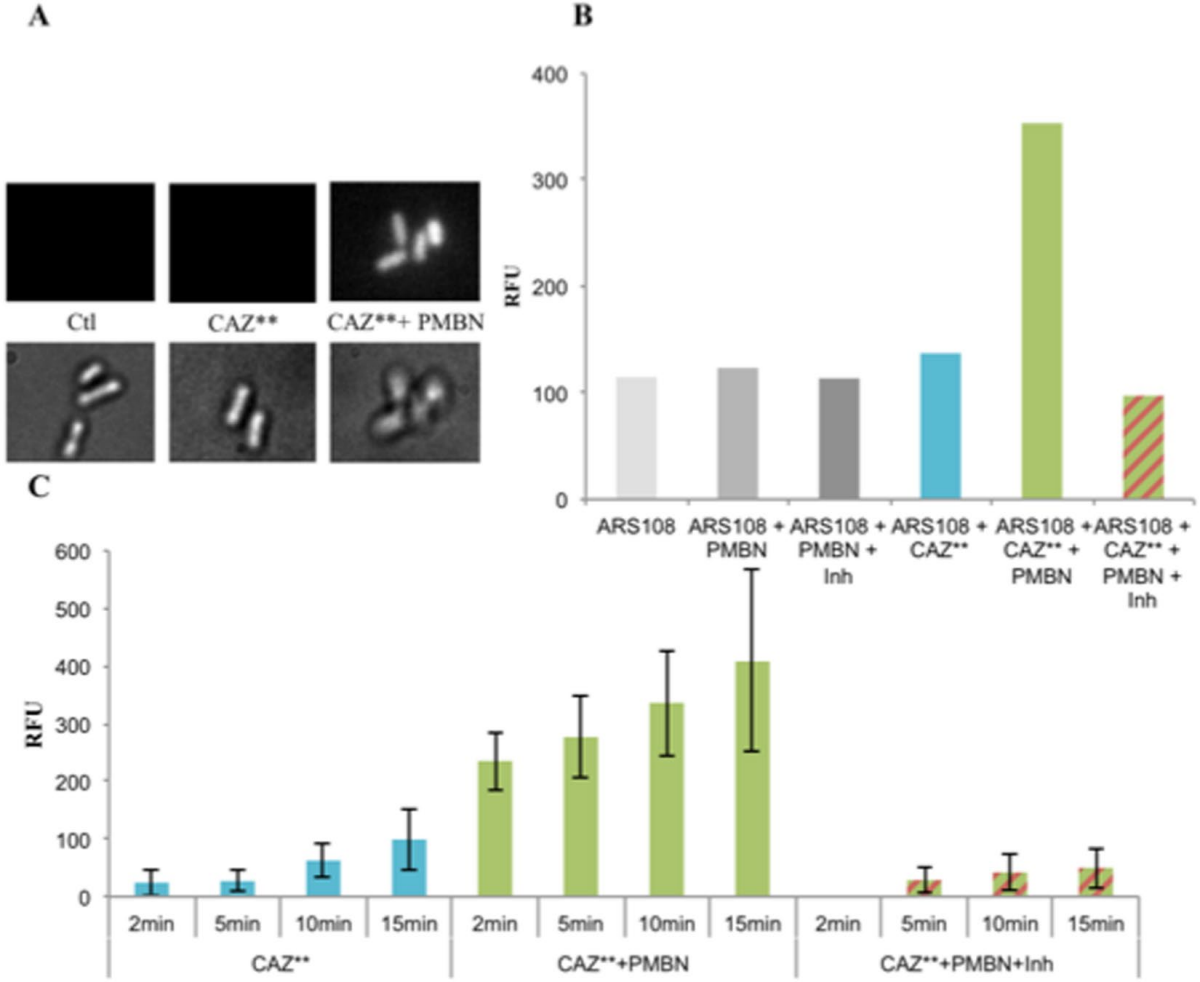
CAZ** accumulation associated with β-lactamase activity in individual bacterial cell devoid of porin. (**A**) Images acquired on DUV microscope. At the bottom, bacteria under visible transmitted light and at the top, the same bacteria excited at 320 nm and emission between 370 and 410 nm. The images showed ARS108 alone (Ctrl), CAZ** with ARS108 and with ARS108 + PMBN at 2 min incubation. (**B**) Fluorescence of CAZ** after 2 min of monitoring corrected by the tryptophan signal. (**C**) Time-courses of CAZ** accumulation: the fluorescence of 6-MeOQ were indicated for the various incubation times and corrected by the tryptophan signal. PMBN: final concentration 102.4 mg/L. Inh: tazobactam + clavulanic acid at 4 mg/L each. The columns with bars (standard deviations) corresponded to measurements carried out with about 40 cells. In all experiments, we measure CAZ** accumulation via the β-lactamase activity that unmasks the fluorescent 6-MeOQ. In the presence of inhibitors (tazobactam + clavulanic acid), the 6-MeOQ release is inhibited. It is important to mention that we cannot detect the β-lactam ring cleavage by PBPs because the turnover of this enzyme is too slow to observe the cleavage product^45^.

Importantly, during the incubation of bacterial strains in the presence of PMBN, no release of ß-lactamase activity was observed in the external medium (Fig. 4S Supplementary Information). This can suggest that PMBN induces an increase of the outer membrane permeability allowing the permeation of small molecules (nitrocefin or ß-lactams), in contrast to polymyxin B that promotes the ß-lactamase release in the same conditions (Fig. 4S Supplementary Information). This indicates that when porins are missing, the addition of PMBN can restore the substrate access to the enzyme located in the periplasmic space.

### Spectrofluorimetry

Two aspects of the CAZ* and CAZ*(S = O) accumulation were investigated, the level of accumulation in the resistant isolate ARS108 that does not synthesize porin, and the role of permeabilizer, PMBN, in the accumulation. To this aim, the ARS108 isolate was incubated alone, with CAZ*/CAZ*(S = O), with the combination CAZ*/CAZ*(S = O) + inhibitors, with the combination CAZ*/CAZ*(S = O) + PMBN, or with the triple combination CAZ*/CAZ*(S = O) + PMBN + inhibitors.

**Figure 5:**
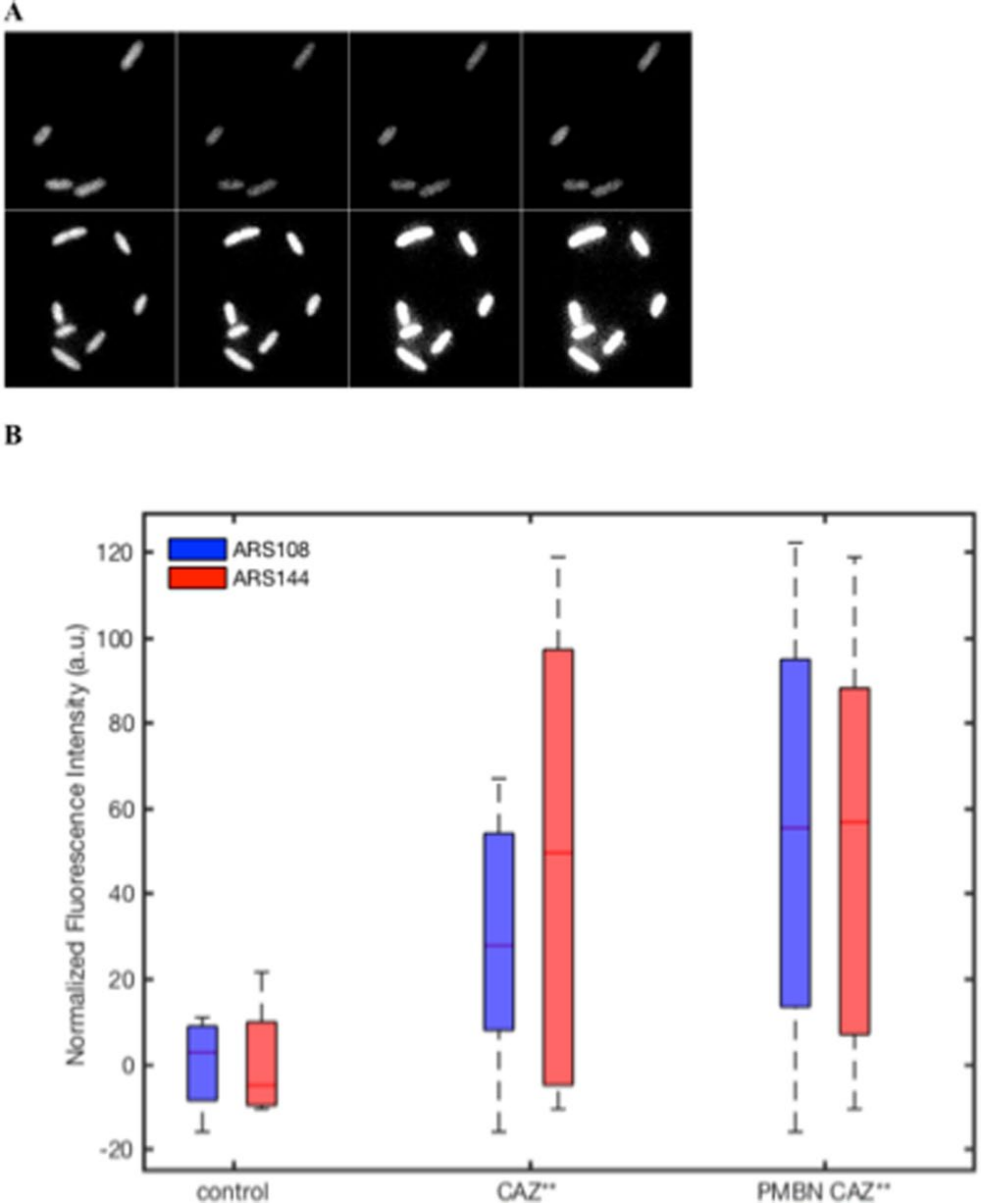
CAZ** accumulation in individual bacterial cell containing porins. (**A**) Images acquired on DUV microscope. The images showed ARS144 alone (upper panel), ARS144 with CAZ** (lower panel) at 6, 12 18 and 24 min incubation respectively. (**B**) CAZ** accumulation measured at 24 min incubation. The 6-MeOQ fluorescence obtained during incubation with the two strains ARS108 (blue) and ARS144 (red) was indicated. The data were corrected by the tryptophan signal and with controls (without CAZ**). PMBN: final concentration 102.4 mg/L. The boxplot corresponded to measurements carried out with about 20 cells.

Firstly, cell-lysates performed on bacterial population were analyzed by spectrofluorimetry and results are presented in Fig. 2. When the incubation was carried out with CAZ*/CAZ*(S = O) alone a weak intracellular accumulation was observed. In contrast, in the presence of PMBN or with the triple combination (CAZ*/CAZ*(S = O) + PMBN + inhibitors), an important increase of fluorescence was obtained suggesting that the membrane permeation is the key step and that PMBN bypasses the porin failure. Interestingly by performing calibration curves (Fig. 5S Supplementary Information), it was calculated that about 2–3 10^−7^ ng of CAZ*/CAZ*(S = O) were accumulated per bacterial cell when the permeability of outer membrane was increased by PMBN (Fig. 2 insert).

Accumulation of CAZ* in intact bacterial cells was visualized by epifluorescence microscopy (Figure 6S Supplementary Information). The fluorescence intensities follow the observations described with lysates. With CAZ* alone, very few labeled bacteria were observed. Addition of β-lactamase inhibitors induced a reduced increase of the fluorescence intensity (this variation was probably due to the biological assay), while incubation in the presence of PMBN + inhibitors led to a six-fold increase. However, this method is not sensitive enough to carry out a more precise analysis on single bacterial cells, so we turned toward the more sensitive microspectrofluorimetry, taking advantage of the possible excitation at 275 nm as previously used^22^.

Figure 3 presented the results regarding the accumulation of CAZ* in individual bacterial cells and indicated an effect of PMBN on the fluorescence intensity. When the signals were normalized relative to tryptophan peak as internal standard^22, 26^, the accumulation was clearly related to the membrane permeabilization by PMBN. In the presence of PMBN we observed a strong increase of CAZ* accumulation compared with the signal obtained during incubation carried out with CAZ* alone (1.2 to 24.5 fluorescence units). In contrast, the addition of inhibitors alone had only a very limited effect on CAZ* accumulation, CAZ* being fluorescent even if it was cleaved by β-lactamases. Regarding CAZ*(S = O), a similar effect was observed on the accumulation level when PMBN was present (Fig. 3 insert).

This suggests that PMBN seriously facilitates the CAZ* uptake inside the porin- strain when the presence of inhibitors has only a minor effect. For the first time, the membranotropic effect of PMBN on the intracellular antibiotic concentration was clearly demonstrated and this result explained previous reports^17^.

Regarding the CAZ** molecule, it is important to mention that this is the 6-MeOQ part resulting from ß-lactamase activity which is fluorescent (Fig. 2S Supplementary Information) and not the original molecule. Consequently, by using CAZ** we can approach two key points: the permeation rate of the CAZ** across the outer membrane and the signal due to periplasmic ß-lactamase activity.

It is demonstrated that CAZ** may efficiently compete with nitrocefin hydrolysis: at equimolar ratio CAZ**/nitrocefin, we observed a 55% reduction of the rate of nitrocefin cleavage (Figure 7S Supplementary Information). This result indicates that CAZ** exhibiting a structure very close to that of CAZ was well recognized and cleaved by the ß-lactamase. In agreement with these kinetics data, the MICs decreased in the presence of *β*-lactamase inhibitors showing that CAZ**, as the parental CAZ, efficiently targets PBPs located in the periplasm (Table 1).

Importantly, after the cleavage of CAZ** by ß-lactamase in the periplasm (as demonstrated for nitrocefin in these conditions Figs 3S and 4S Supplementary Information), the resulting 6-MeOQ (a small molecule MW: 159.18 g/mol) was released in the supernatant fraction during the centrifugation (Figure 8S Supplementary Information). Consequently, to correlate the CAZ** accumulation with the fluorescence signal reflecting the periplasmic cleavage, we recorded its by time-course accumulation in intact individual bacterial cells (Fig. 4).

We can conclude that CAZ** accumulation was maximal in the presence of PMBN that boosts the permeation rate in the strain devoid of porin (Fig. 4A). In Fig. 4B the various accumulations were presented after 2 min of incubation under various conditions. The role of the outer membrane barrier was clearly shown and, importantly, the relationship between the measured signal and ß-lactamase activity was documented demonstrating the correct location of CAZ** in the periplasmic space where it was cleaved by the enzyme in the absence of ß-lactamase inhibitor generating 6-MeOQ (Fig. 4). The kinetic analyses of CAZ** accumulation reported in Fig. 4C reflected the intra-bacterial accumulation that depends on the outer membrane translocation. When the assay was performed in the porin producer strain (ARS144), we observed a rapid penetration and accumulation of CAZ**, no significant variation was obtained in the presence of PMBN (Fig. 5). A maximum was obtained at 24 min incubation time.

In the strain devoid of porin, we observe a fast penetration rate during the first two minutes followed by a slower accumulation rate for long incubation times (Fig. 4, Figure 9S Supplementary Information). In contrast, in the absence of permeabilizer, a weak accumulation curve was observed. The ratio of CAZ** accumulation obtained in the presence to those in the absence of PMBN was comparable to the ratio obtained with CAZ* (Figs 3 and 4). It is noted that PMBN restores CAZ** accumulation in the porin- bacterial cells (e.g. ARS108) at a level similar to ones observed in normal porin producer strain (e.g. ARS144) (Figs 4 and 5).

## Discussion

Faced with the tidal wave of multidrug resistance, it is urgently required to determine the intracellular accumulation of antibiotic molecules in order to correlate the concentration to the antibacterial activity. In some case, the intrinsic fluorescence of antibiotics has been used to follow the intra-bacterial accumulation such as previously reported for ciprofloxacin or fleroxacin in MDR clinical strains^22, 26^.

Interestingly, ceftazidime is now proposed in combination with ß-lactamase inhibitors^12, 14–16^ for the treatment of ß-lactamase-producing *Enterobacteriaceae*, an emerging risk documented by a recent ECDC report (http://ecdc.europa.eu/en/publications/Publications/antimicrobial-resistance-europe-2014.pdf). The uptake of CAZ may be studied using our conditions in order to evaluate the influx and the stability of CAZ in the periplasmic space of ESBL-producing *Enterobacteriaceae*. Moreover, the distribution of cephalosporin concentration can be studied in bacterial population in order to determine the possible heterogeneity of the intracellular labeling as previously reported for fluoroquinolone in MDR strains^26^.

Regarding the ß-lactam family, the production of ß-lactamases in addition to the membrane barrier due to the downregulation of porin expression (including also the alteration of porin function), strongly contributes to the decrease of the periplasmic concentration of the antibiotic under the threshold required to inhibit bacterial growth (for reviews see refs 8, 9, 27 and 28). Thus, it is necessary to understand the role of the membrane permeability and the periplasmic ß-lactamase on the bacterial susceptibility to clinically-used ß-lactams such as CAZ in resistant clinical strains. To this aim, we have developed several CAZ-probes in order to enlighten the permeation rate, the periplasmic accumulation and the hydrolysis activity in selected clinical strains. The study of these parameters on the same samples supports the possibility to detect active molecule close to its target in MDR isolate and pave the way of original combination to bypass the mechanical and enzymatic barrier used by resistant bacterial cells.

Two series of fluorescent CAZ derivatives have been designed and synthesized. The signal is only observed in periplasm when the PMBN permeabilizes the outer membrane barrier as correlated by the activity measured in ARS108 clinical strain. When we analyzed the various CAZ* intracellular levels, we obtained a very low penetration in the strain devoid of porin in accordance with the susceptibility. In the presence of PMBN that renders the outer membrane more permeable^17, 23^ and stimulates the CAZ* influx, we observed an increase of susceptibility suggesting a saturation of enzymatic barrier in the resistant strain. Interestingly, we also obtained a restoration of CAZ activity in the presence of inhibitors, but the susceptibility was fully restored by the combination PMBN + inhibitors.

This bypass of the membrane barrier is clearly illustrated both by the increase of accumulation level obtained in these conditions and of susceptibility observed on the resistant isolate. The respective actions of PMBN and polymyxin B on the outer membrane barrier are clearly reported and fit in well with the previous publications reporting a significant increase of the ß-lactam activity in strains devoid of porins with polymyxin combination^29–32^. Interestingly, PMBN-facilitating role in ß-lactam translocation is also observed in the presence of porins demonstrating that the porin channel exhibits some steric/charge constraints that limit drug diffusion^33–36^.

For the first time, a correlation between internal accumulation and ß-lactamase activity is provided that supports the pioneer model proposed by H. Nikaido^27, 37^. CAZ** also illustrates the effect of periplasmic ß-lactamase on the CAZ concentration close to the target, this represents an approach of the intrabacterial concentration required to obtain an antibacterial effect on resistant strains. In addition, CAZ** directly enlightens the correlation between periplasmic accumulation and activity: the fluorescence signal intensity obtained only after the periplasmic cleavage of CAZ** is reflected by the MIC evaluated without or with ß-lactamase inhibitor (compare Table 1 and Figs 4 and 5). Moreover, the kinetics of CAZ** cleavage reflects a fast permeation rate for CAZ** in the presence of PMBN (Figs 4 and 9S).

CAZ** being an efficient competitor of nitrocefin hydrolysis and presenting a significant antibacterial activity (compare to CAZ), it could be interesting to test it in competition with other ß-lactams to evaluate the respective accumulation rate (and affinity for ß-lactamase) and, by this way, propose a diffusion ranking. Moreover, it can be used to determine the permeation rate through the OmpC and OmpF channels *in vivo*: it is important to evaluate the *in vivo* respective translocation rate in various *Enterobacteriaceae* strains producing different porins (OmpC, OmpF, OmpN, PhoE) since *in vitro* data, using purified porins and electro-physiological approaches, reported some differences between porins and ß-lactams^38–40^. In the clinical strain that overexpressed ß-lactamase and contained porins, the accumulation is quite similar to the porin- strain treated with PMBN. This reflects the important role of porins during cephalosporin uptake and with the previous reports mentioning the channel differences in the OmpF and OmpC porins, it will be interesting to assay fluorescent cephalosporins in the different channel backgrounds.

Studies are now under current investigation for trying to correlate the killing rate in strains expressing diverse outer membrane channels to the intracellular concentration of specific antibiotics.

This is key information necessary to approach the “Structure Intracellular Concentration Activity relationship” (SICAR), a new concept for the future antibiotic candidate that will correlate permeation parameters to activity properties for a defined molecule^41^. The key role of outer membrane permeability in the accumulation of cephalosporin molecules, and by extension of those of the ß-lactam family, in the periplasmic space can be now quantified. This opens the way for a new definition of the real concentration necessary to inhibit bacterial growth as discussed in IMI Translocation consortium^7^. Moreover, the permeation kinetics and the location of the ß-lactam in the periplasmic space become a future objective in order to correlate, in real time, the local concentration of antibiotics close to the targets with a more effective drug design.

## Methods

### Chemicals and synthesis

The structures of CAZ* and CAZ*(S = O) as well as CAZ** are depicted in Fig. 1. The syntheses, complete procedures and characterizations are detailed in the Schemes S1–S3 **(**Chemistry Supplementary Information).

### Bacterial strains

*Escherichia coli* strains and clinical isolates, used in this study are listed in Table 1. These strains have been previously characterized for their porin and ß-lactamase contents^17, 23, 42^.

### Drug susceptibility assays

Ceftazidime (CAZ) was used to assess the antibiotic susceptibility of *E. coli* strains. Polymyxin B nonapeptide (PMBN) and inhibitors were used to assess the contribution of the membrane and enzymatic barrier in antibiotic activity (porin absence, ß-lactamase). MIC values of antibiotics were determined by the microdilution method (CLSI) in liquid Mueller Hinton II media by using the twofold standard microbroth dilution method (microplates and automatic analyses Tecan®) (CLSI, http://clsi.org/). An inoculum of 2.10^5^ CFU (colony-forming-unit) in 200 µL of broth containing 2-fold serial dilutions of each antibiotic was used in the absence or presence of permeabilizer (PMBN used at sub-inhibitory concentration) or ß-lactamase inhibitors (tazobactam and clavulanic acid at 4 mg/L each) as previously described^17, 23, 39^. MIC values were read after 18 h of incubation at 37 °C. Values are medians of at least three independent experiments and are presented in mg/L.

### Nitrocefin assay for measurement of β-lactamase

β-lactamase activity with nitrocefin as substrate was determined by measuring the product of nitrocefin (Oxoid^®^) hydrolysis at 490 nm. Strains grown to exponential phase were collected, pelleted, and resuspended in buffer before disruption if necessary. Specific activity was defined as nitrocefin hydrolyzed/min/mg protein determined using the initial near-linear slope of the curve and with protein measured by the BCA method in the sample^17^.

### Nitrocefin assay for ß-lactamase release

To evaluate if PMBN can permeabilize the outer membrane and promote the release of ß-lactamase, the enzymatic activity was measured in bacterial cell and in external medium. Suspensions of ARS108 or ARS144 strains were incubated 15 min at 37 °C in the absence or in the presence of PMBN (102.4 mg/L), or polymyxin B (102.4 mg/L) used as control. After centrifugation of bacterial suspensions, 6,000 × *g* (15 min at 4 °C), the supernatant corresponding to the incubation medium was assayed for its ß-lactamase content. The bacterial cell pellet was frozen, then resuspended in an equivalent volume of deionized water containing lysozyme (0.5 mg/L) and polymyxin B (280 mg/L). Then, two runs of cell disruptor (ConstantSystems Ltd, Northants, UK) at 2kBar were performed to lyse the intact bacteria. The resulting lysate was centrifuged at 9,000 × *g* (15 min at 4 °C) and the supernatant that corresponds to the whole cell lysate was used for ß-lactamase assay as described above.

### Inhibition of nitrocefin hydrolysis by CAZ**

Bacterial cells were collected at exponential grown phase in Luria-Bertani broth (10 g tryptone, 5 g yeast extract, 10 g NaCl, per L; pH 7.5), pelleted, and resuspended in water before sonic disruption. Assays were performed on the sonicated supernatants. Nitrocefin hydrolysis was monitored for 30 min in a sodium phosphate buffer (50 mM, pH 7) and 25 µL of ARS108 lysates were used in a total volume of 200 µL. Three different conditions were performed: 1) bacterial lysates with nitrocefin (48 µM); 2) bacterial lysates with nitrocefin (48 µM) and β-lactamases inhibitors (tazobactam and clavulanic acid at 4 mg/L each); 3) bacterial lysates with an equimolar mix of nitrocefin and CAZ** (48 µM each). The ß-lactamase activity was determined by the slope of the tangent as described above^17^.

## Determination of the intracellular accumulation of CAZ

### Bacterial lysates

Bacteria grown at 37 °C in Luria-Bertani broth to mid-exponential-phase (corresponding to 0.6 optical density units at 600 nm) were concentrated 10-fold. Briefly, the bacterial suspension was centrifuged at 6,000 × *g* (15 min at 20 °C) and pellets were re-suspended in 1/10 of the initial volume in a sodium phosphate buffer (50 mM) at pH 7 supplemented with MgCl_2_ (5 mM) (NaPi-MgCl_2_ buffer) to obtain a density of 6.10^9^ CFU.mL^−1^. 1.6 mL of the bacterial suspension was incubated for 30 min at 37 °C (final volume 2 mL) with each compound: CAZ*, CAZ*(S = O) and CAZ** at 32 mg/L, in the absence or in the presence of Polymyxin B nonapeptide (PMBN) at 102.4 mg/L and with and without inhibitors (tazobactam and clavulanic acid) at 4 mg/L each. Bacterial suspensions incubated without antibiotics, with or without PMBN, and without or with inhibitors were used as controls. The same accumulation sample was used to measure fluorescence with spectrofluorimeter and microscopy. Suspensions (800 μL for spectrophotometer measurement or 400 μL for microscopy) were then loaded on 1 M sucrose cushions (1,100 μL or 550 μL respectively) and centrifuged at 9,000 × *g* for 5 min at 4 °C to eliminate extracellular-adsorbed compounds and collect washed bacteria. After centrifugation, pellets corresponding to 800 μL of bacterial suspensions were lysed with 500 μL of 0.1 M Glycin-HCl pH 3 buffer for 2 h at room temperature. After a centrifugation for 15 min at 9,000 × *g* and 4 °C, 400 μL of lysates were diluted in 600 μL of NaPi-MgCl_2_ buffer and analyzed by spectrofluorimetry (Ex 275 nm, Em 420–480 nm for CAZ* and CAZ*(S = O)^22, 26^. Briefly, the fluorescence spectra of bacteria lysates were recorded at 20 °C using a FluroMax-4 (HORIBA Jobin Yvon INC, Chilly Mazarin, France) spectrofluorimeter and a 0.5 cm pathlength quartz cuvette for measurement. Fluorescence emission spectra of lysates were recorded at an excitation wavelength suitable for compounds detection (*e.g*. in 0.1 M buffer of Glycin-HCl of pH = 3)^22, 26^. To quantify the compound fluorescence intensity in bacteria lysates, spectra were normalized using the tryptophan peak at 356 nm before subtraction of control spectra (no compound, with or without PMBN). Compound concentrations in bacteria lysate were calculated according to a calibration curve generated by mixing a known concentration of each compound with bacterial lysate and measured with spectrofluorimeter (n = 3).

To control that the bacterial cells are alive during the experimental time, we determined the number of CFU by sampling the bacterial suspension at 30 min during antibiotic incubation. CFUs were determined along the experiment and no change in cell viability was observed during this period that corresponds to accumulation assay (data not shown). It must be noted that the ratio bacterial cell/antibiotic concentration were different in MIC assays and in accumulation assays due to the threshold necessary for signal detection with the conditions previously described^22, 26^.

### Epifluorescence microscopy

Bacteria were incubated with CAZ* (32 mg/L) for 30 min at 37 °C with or without ß-lactamase inhibitors (tazobactam and clavulanic acid, 4 mg/L each) and with or without PMBN (102.4 mg/L) as described in a). To pellets corresponding to 400 μL of bacterial suspensions (DO_600_ = 4.8) were added 200 μL of sterile NaPi 50 mM pH 7. A drop of 2 μL was mounted on agarose slides prepared as previously reported^43^. Mounted slides were scanned with an epifluorescence microscope Nikon Eclipse TE-2000 (Nikon Instruments Europe, Badhoevedorp, The Netherlands) using a 100x objective and a DAPI cube (BP 360/40). Image analysis and the raw intensity of fluorescence were performed and measured using the ImageJ software (NIH, Bethesda, MD, USA).

### Deep ultraviolet (DUV)-Fluorescence localization

To detect the CAZ* and CAZ*(S = O) fluorescence from single bacteria background, pellets corresponding to 400 μL of bacterial suspensions were re-suspended in 100 μL of NaPi-MgCl_2_ buffer. 0.5 µL of resuspended pellets were deposited between two quartz coverslips and analysed by DUV fluorescence imaging (Ex 372 and Em 420–480 nm) at DISCO Beamline^44^. UV epifluorescence microscopy focalises synchrotron ultraviolet beam under 350 nm through an all quartz microscope in order to benefit from a broader range of native fluorophores and an increased lateral resolution directly related to the shorter excitation wavelengths.

We tested bacterial strains with CAZ* or CAZ*(S = O) in the absence or presence of PMBN, in the absence or presence of inhibitors, and in the presence of PMBN + inhibitors with the incubation time of 30 min. Recorded images were treated as previously described^22^.

For time-course accumulation in individual bacteria, the intracellular accumulation was monitored directly under the DUV microscope. Bacteria were concentrated in order to obtain an OD of 4.8 in sterile NaPi-MgCl_2_ buffer (NaPi 50 mM pH 7). Then 120 µL of the suspension were centrifuged on a 1 M sucrose cushion (165 μL) at 9,000 × *g* for 5 min at 4 °C. The pellets were resuspended in 40 µL NaPi-MgCl_2_ buffer containing or not molecules (CAZ** 32 mg/L; PMBN 102.4 mg/L; tazobactam and clavulanic acid, 4 mg/L each). Resuspended pellets (0.5 µL) were immediately deposited between two quartz coverslips and followed by DUV fluorescence imaging at excitation wavelength of 320 nm and emission between 370 and 410 nm (for CAZ**) and between 327 and 353 nm for tryptophan. Recorded images corresponding to the incubation times were exported and treated. The strain ARS144 that produced OmpC and OmpF was used to compare the CAZ** accumulation when porins were produced.

## Electronic supplementary material

SUPPLEMENTARY INFORMATION
