# Supplementary material for: Microspectrofluorimetry to dissect the permeation of ceftazidime in Gram-negative bacteria
Source: Sci Rep. 2017 Apr 20;7:986. doi: 10.1038/s41598-017-00945-8 (PMC5430551; doi:10.1038/s41598-017-00945-8)

SUPPLEMENTARY INFORMATION

Microspectrofluorimetry to dissect the permeation of ceftazidime in Gram-negative bacteria

Anas Allam[1,*], Laure Maigre[2,*], Julia Vergalli[2,*], Estelle Dumont[2,*], Bertrand Cinquin[3], Rodolphe Alves de Sousa[1], Jelena Pajovic[3], Elizabeth Pinet[2], Nikaia Smith[1], Jean-Philippe Herbeuval[1], Matthieu Réfregiers[3], Isabelle Artaud[1,§], Jean-Marie Pagès[2,§]

SUPPLEMENTARY FIGURES

Table 1S: Bacterial susceptibility (in mg/L) to modified-ceftazidimes

	AG100*	AG100A*	ARS108#
**Porin**	+	+	-
**AcrAB efflux pump**	+	-	+
**ß-lactamase**	AmpC basal	AmpC basal	AmpC CTX-M-15
**CAZ (S=O)**	2	1	8
+ PMBN	0.25	0.125	0.25
+ Inh	1	nd	4
+ Inh + PMBN	0.25	0.125	0.25
**CAZ*(S=O)**	64	16	>64
+ PMBN	8	8	8
+ Inh	32	16	64
+ Inh + PMBN	8	nd	4

*,# Isogenic strains [AG100 (parental) and AG100A, AcrAB- derivative] and clinical isolates [17,24,43].

Porins and efflux components identified by Western Blot-immunodetection (OmpC or OmpF, AcrAB): -, no signal (*e.g.* no OmpC, no AcrAB).
CAZ (S=O), ceftazidime oxidized form; CAZ*(S=O) fluorescent derivative.
PMBN final concentration 51.2 mg/L for AG and 102.4 mg/L for ARS strains
Inh: tazobactam + clavulanic acid, 4 mg/L each.
nd, not determined
Values are medians of at least three independent experiments and are presented in mg/L.

Figure 1S. Fluorescence and UV-vis Spectrum of CAZ*, CAZ*(S=O), CAZ**

1) CAZ*: 10 μM in NaPi 50 mM pH 7.0; data fluo: 700V, split Ex EM 2.5, 2.5

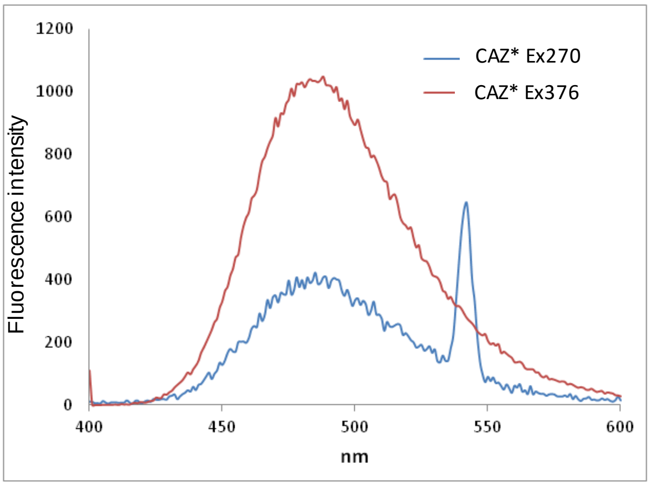

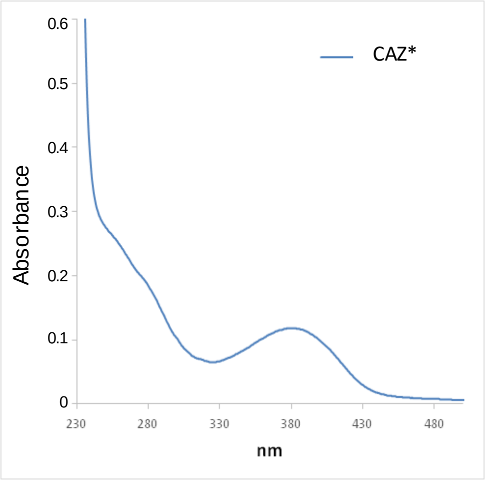

2) CAZ*(S=O): 10 μM in NaPi 50 mM pH 7.0; data fluo: 950V, split Ex EM 2.5, 2.5.

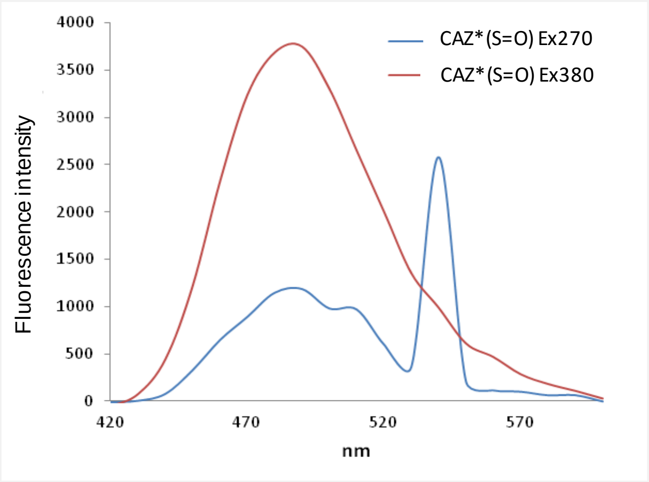

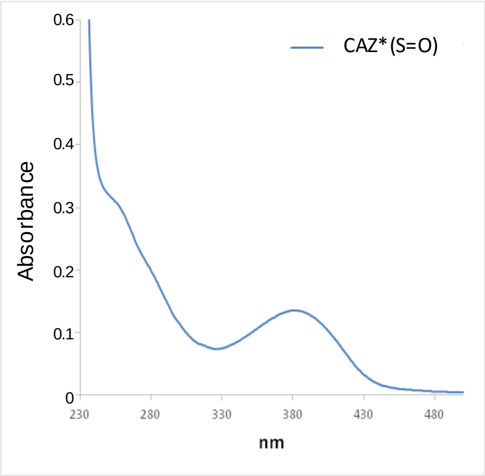

3) CAZ**: 10 μM in NaPi 50 mM pH 7.0; data fluo: 600V, split Ex EM 5.5.

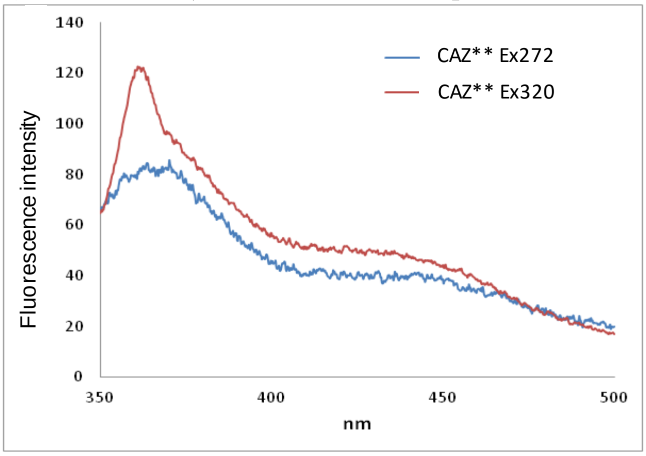

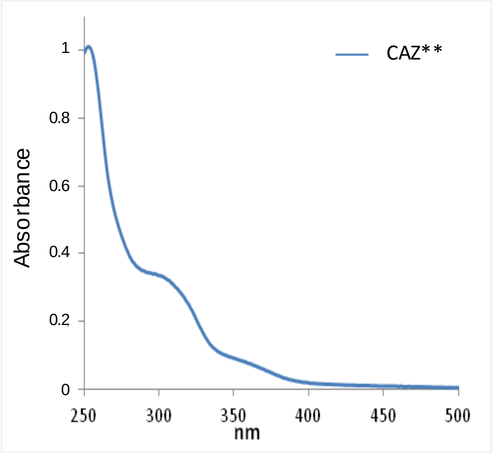

Figure 2S: Product release by β-lactamase from CAZ (6-MeOQ):** 10 µM in NaPi 50 mM pH 7.0; data fluo: 600V, split Ex EM 5.5

A) β-lactamase activity

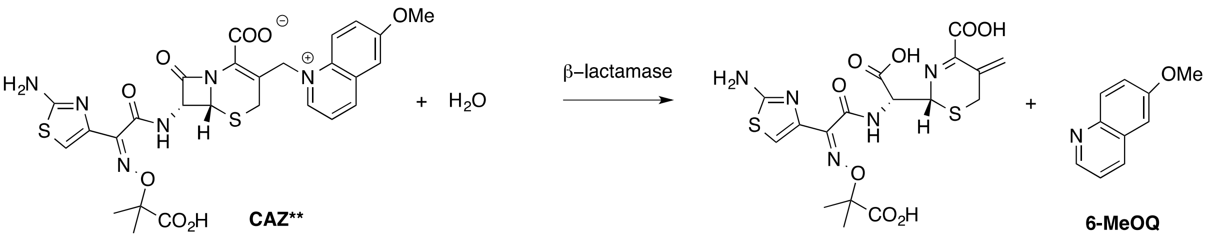

B) Spectroscopic characterization of 6-MeOQ

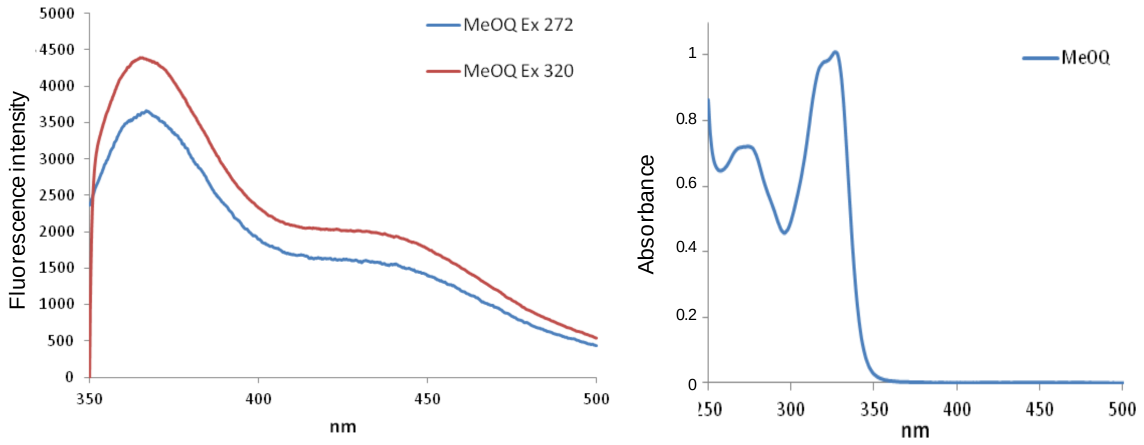

After cleavage by periplasmic ß-lactamase, the release of 6-MeOQ was detected in UV at 330 nm and by fluorescence upon excitation at 320 nm and emission at 370 nm.

Figure 3S: Nitrocefin hydrolysis and outer membrane permeation.

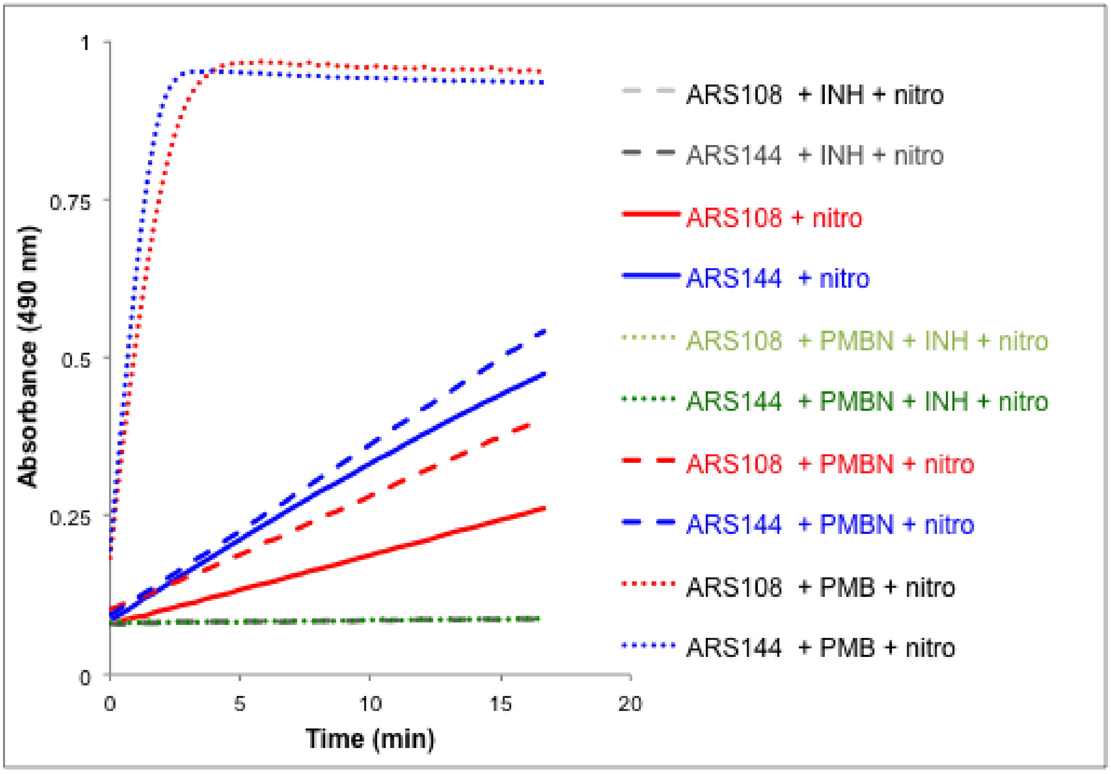

Strains/conditions	mU/min/mg prot
ARS108	0.3 ± 0.02
ARS108+PMBN	0.6 ± 0.03
ARS108+PMBN+INH	0.006± 0.0008
AS144	0.7± 0.025
ARS144+PMBN	0.8± 0.035
ARS144+PMBN+INH	0.013± 0.0012

Standardized suspensions (100 µL corresponding to 0.6 absorbance unit at 600) of ARS108 or ARS144 strains in NaPi buffer were incubated at 37°C with nitrocefin (50 mg/L equivalent at 97 µM). The incubations were performed in the absence or in the presence of PMBN (102.4 mg/L), in the absence or in the presence of inhibitors (tazobactam and clavulanic acid, 4 mg/L each), Incubations with polymyxin B (102 mg/L) were presented as control.
The nitrocefin hydrolysis was monitored at 490 nm: ARS108 (porin-); ARS144 (porin+).
Incubations were performed:

in the absence (intact lines) or in the presence of PMBN (dotted lines)
in the presence of PMBN + inhibitors (dotted green lines)
in the presence of inhibitors (dashed grey lines)
in the presence of polymyxin B (dotted lines)

Values are means of at least three independent assays, the error bars are not included in the graph for clarity.

Figure 4S: Localization of ß-lactamase during the incubation of *E. coli* strains carried out in the absence or presence of membrane permeabilizers

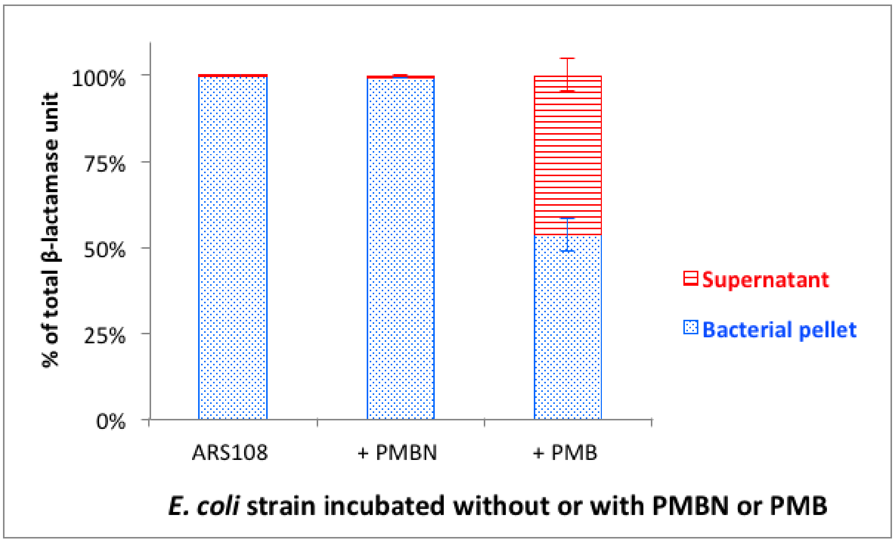

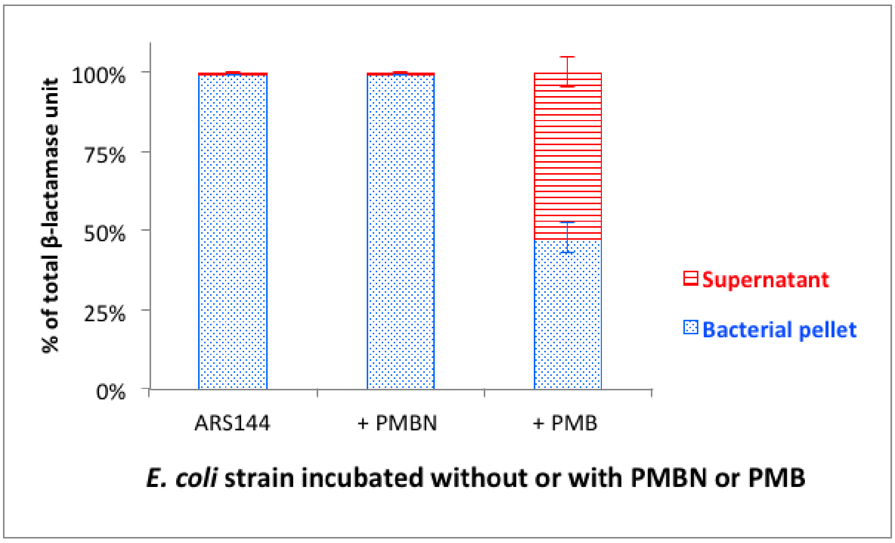

Suspensions of ARS108 or ARS144 strains were incubated 15 min at 37°C in the absence or in the presence of PMBN (102.4 mg/L) or polymyxin B (102.4 mg/L). After incubation, the suspension was centrifuged 15 min at 6,000 x *g* and 4°C. The supernatant corresponding to incubation medium was used for ß-lactamase assay. Bacterial cell pellet was frozen, then resuspended in deionized water containing 0.5 mg/L of lysozyme and polymyxin B (280 mg/L) and two runs of cell disruptor (ConstantSystems Ltd, Northants,UK) at 2 kBar. The lysate was centrifuged for 15 min at 9,000 x *g* and 4°C. Supernatant corresponding to total cell lysate was used for ß-lactamase assay.
The ß-lactamase activity determined in the two fractions was plotted as % of the total enzymatic activity. Values are means of at least three independent assays.

Figure 5S: Calibration curves: standard curves of CAZ* and CAZ*(S=O) fluorescence intensity.

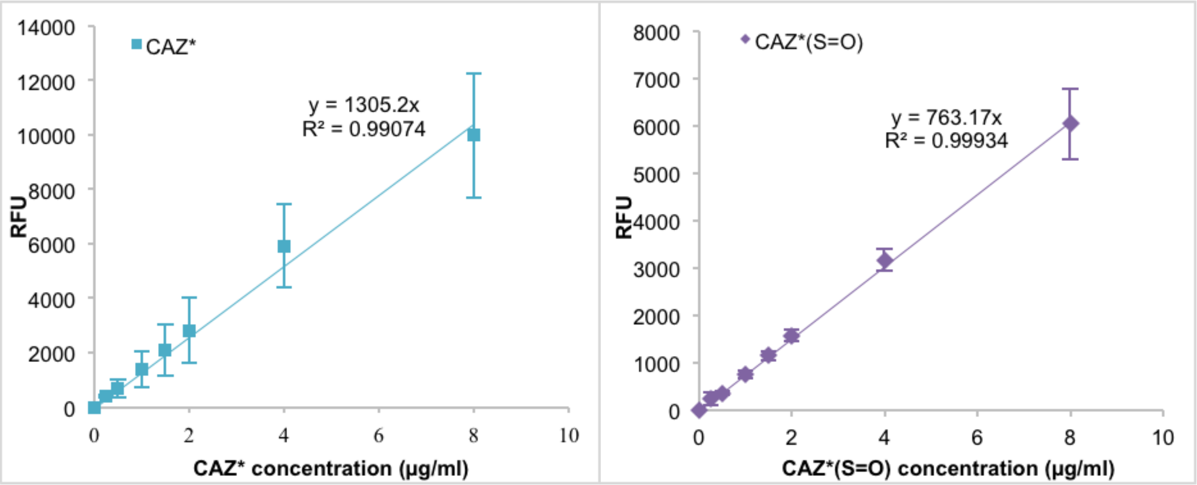

Various concentrations of CAZ* and CAZ*(S=O) were mixed with bacteria lysates at OD_{600}= 4.8 and measured with spectrofluorimeter (n=3).
Excitation wavelength 275 nm, emission peak measured at 450 nm.

Figure 6S: Microepifluorescence analyses of CAZ* accumulation

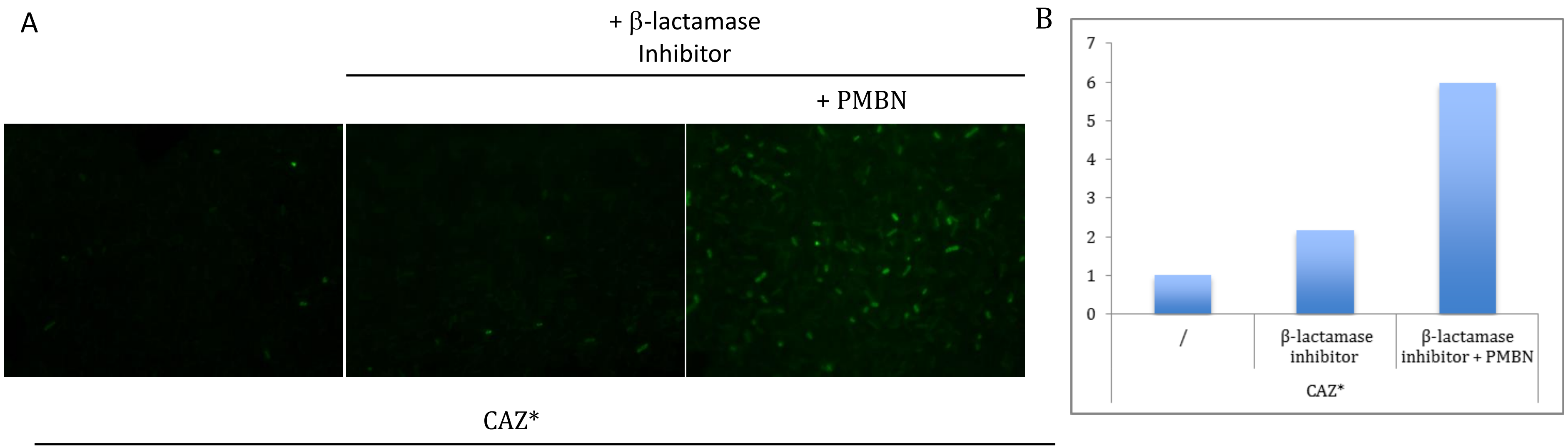

Bacteria ARS108 were incubated for 30 min at 37°C with CAZ* (32 mg/L) with or without ß-lactamase inhibitors (tazobactam and clavulanic acid, 4 mg/L each) and with or without PMBN (102.4 mg/L) to increase bacteria permeability then mounted on agarose slides.
A, The panels show confocal microscopy images of the fluorescence of CAZ* in the different conditions using the DAPI Cube.
B, The graph represents the intensity of fluorescence of each condition normalized to CAZ* alone.

Figure 7S: Competition of the hydrolysis rate of Nitrocefin with CAZ**

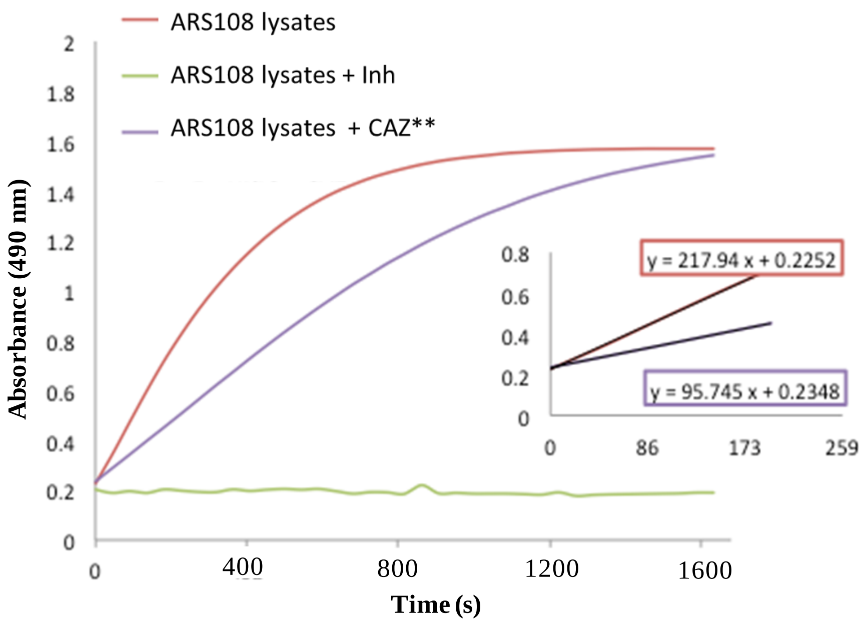

Monitoring of the nitrocefin hydrolysis at 490 nm. Nitrocefin hydrolysis was performed with ARS108 lysates (red line), with ARS108 lysates + β-lactamases inhibitors (green line), with ARS108 lysates + CAZ** (purple line). Nitrocefin concentration: 48 µM; Inhibitors: tazobactam and clavulanic acid (4 mg/L each); CAZ** concentration: 48µM.

Insert: Extrapolation of the rate of nitrocefin hydrolysis by the slope of the tangent at the intercept.

Fig 8S: Detection of 6-MeOQ in the supernatant after 30 min of incubation

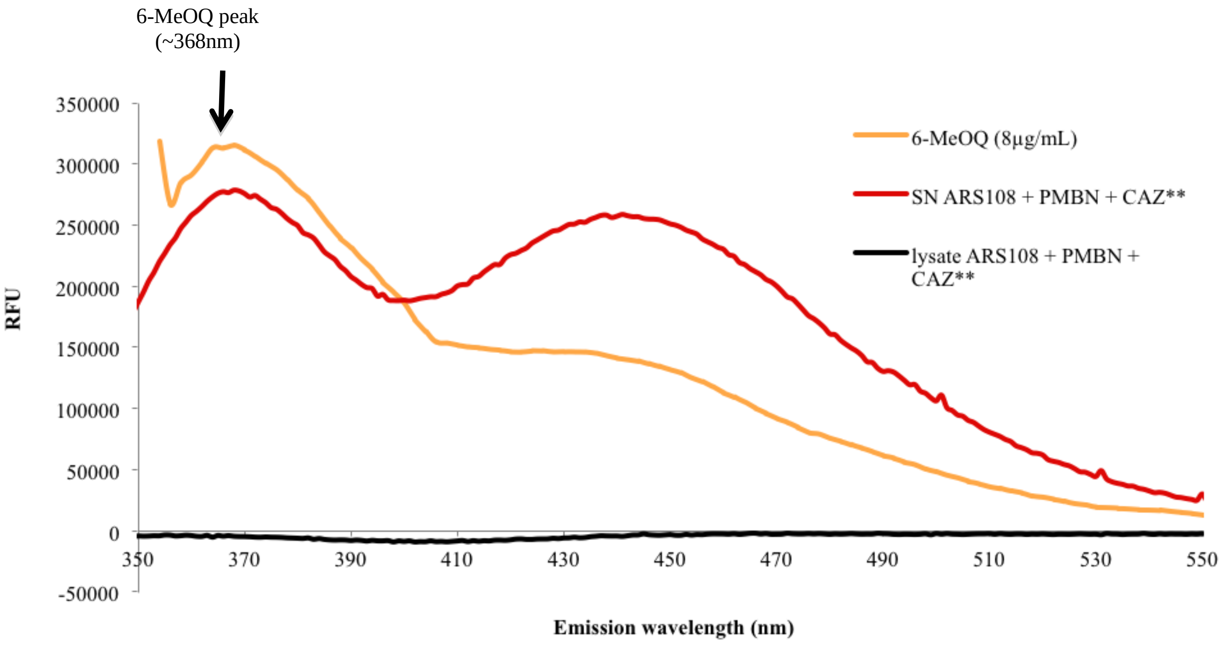

Suspension of ARS108 strain was incubated 30 min with CAZ**. Bacterial suspensions were loaded on sucrose cushions and centrifuged to eliminate extracellular-adsorbed compounds and collect washed bacteria. After centrifugation, the supernatant (SN) was kept and the cells pellet was lysed. Fluorescence in supernatant and lysate was measured (excitation wavelength 320 nm).

The emission peak corresponding to the 6-MeOQ (orange line) was observed around 368 nm. The red line indicated the amount of the fluorescent product (6-MeOQ) due to the hydrolysis of CAZ** by β-lactamase and external release in the first centrifugation supernatant after subtraction of the background. The black line indicates the fluorescent signal in the cell lysate obtained from the cells pellet following the first centrifugation, after subtraction of the background.

SN: supernatant after the first centrifugation performed to separate the bacterial intact cells to the incubation medium.

Figure 9S: Kinetics of CAZ accumulation in ARS108 determined by fluorimetric detection of the fluorescence side chain after ß-lactamase cleavage**

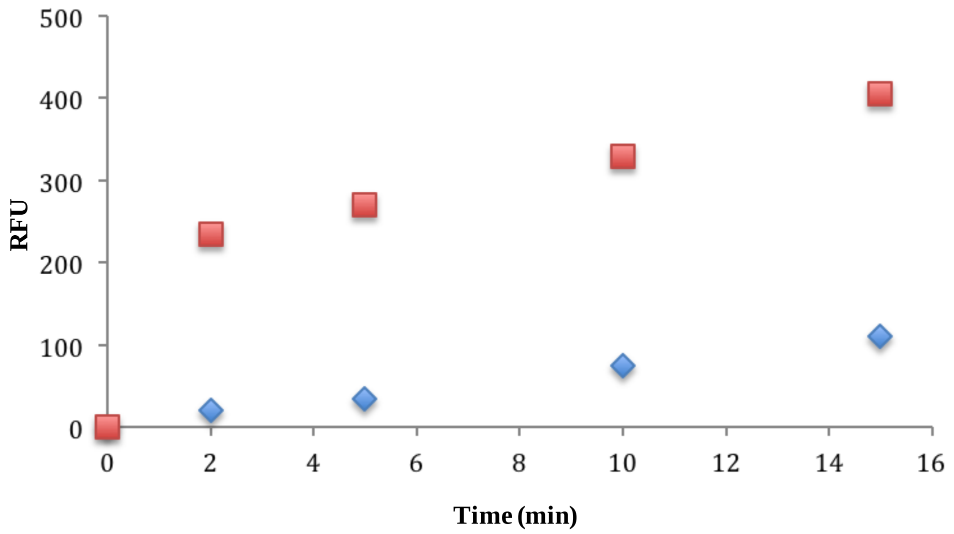

Images acquired on DUV microscope at various incubation times of ARS108 with CAZ**. The fluorescence of CAZ** was indicated for the various incubation times and corrected by the tryptophan signal.
Blue diamonds and red squares correspond to the signals obtained (see Figure 4) with CAZ** alone and CAZ** in the presence of PMBN respectively.

SUPPLEMENTARY CHEMICAL INFORMATION

Chemistry

All solvents and chemicals were purchased from SDS and Aldrich, respectively. Ceftazidime was purchased from TCI Europe and dry DMF from Aldrich. CH_2Cl_2 was dried using a standard procedure. ^{1}H NMR spectra were recorded on a Bruker ARX-250 or Bruker AvanceII-500 spectrometer and chemical shifts were reported in ppm downfield from TMS. Electrospray ionization (ESI) mass spectrometry analyses were recorded on a Thermofisher Exactive (orbitrap) high resolution spectrometer. Elemental analyses were carried out by the microanalysis service at Gif-sur-Yvette CNRS.

Although some fluorescent cephalosporins have been previously synthezised to target ß-lactamases,[1,2] the present objective was to prepare a labeled ceftazidime whose activity is close to that of the parent compound and whose addressing inside the bacteria can be monitored by microspectrofluorimetry.

1. Stereoselective synthesis of fluorescent ceftazidimes CAZ* and CAZ*(S=O)

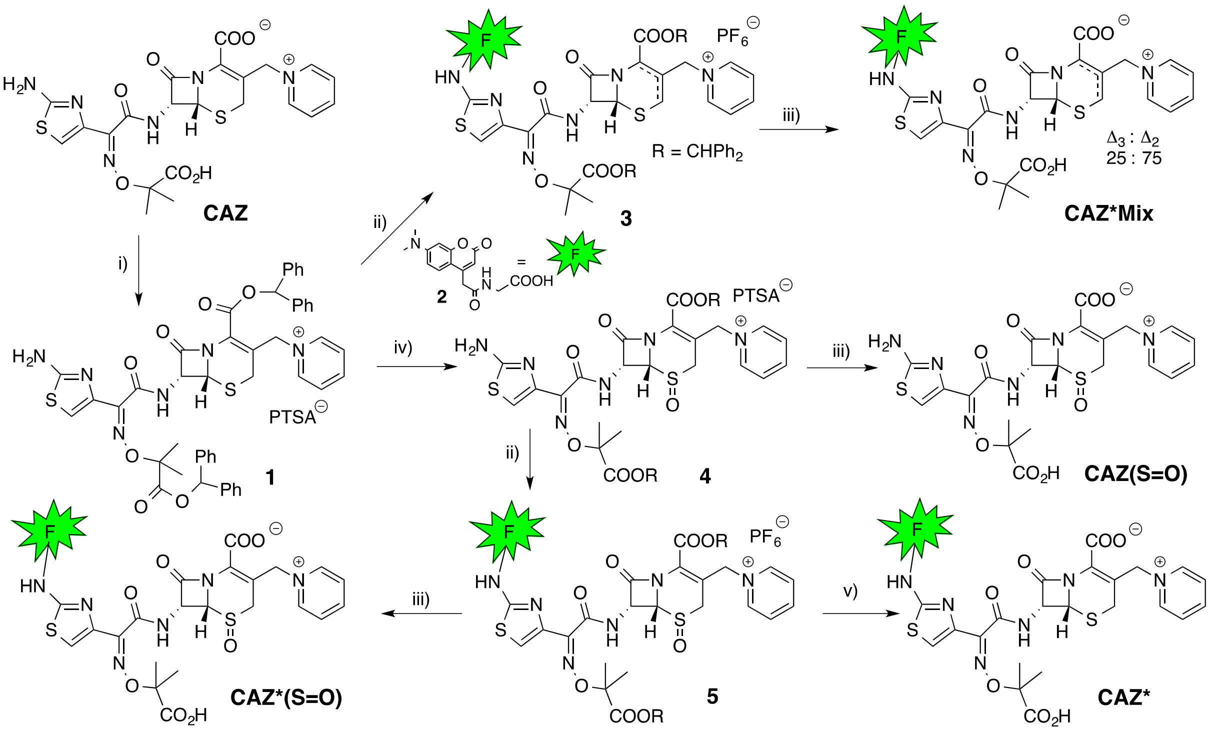

Scheme S1: Synthesis of fluorescent cephalosporin under its reduced form **CAZ*** and oxidized form **CAZ*(S=O)**

i) Ph_2CN_2, PTSA, CH_3CN / H_2O ii) HOAT / HATU, NMM, DMF **2** iii) TFA / CH_2Cl_2, H_2O TIS iv) *m*CPBA CH_2Cl_2 0°C r.t. v) a) PCl_3 DMF, -10°C b) TFA / CH_2Cl_2, H_2O TIS

We first chose to introduce the coumarin fluorescent probe at the level of the amine of the aminothiazol via a glycyl spacer. The synthesis started from commercial ceftazidime as depicted in Scheme S1. The free carboxylates were first converted into the benzhydryl esters **1** with Ph_2CN_2. The first strategy was to couple 7-dimethylaminocoumarin-4-acetyl glycine with **1** to afford **3** and then to deprotect the acids. During the coupling reaction, an isomerization of the double bond of the cephem ring from the Δ3 to the Δ2 position occurred leading to CAZ*Mix. Indeed, this tendency of the double bond to migrate to the more stable Δ2 position, even under slightly basic conditions, is a problem frequently encountered in cephem chemistry[3] that has to be solved since the only active form of cephalosporin is the Δ3 one. Reconjugation with the carboxylate requires sulfur oxidation and further reduction. To get exclusively the Δ3 isomer of CAZ* we tried sulfur oxidation of **3** with *m*CPBA in CH_2Cl_2 as previously described by Kaiser et *al.*[4] However due to the low solubility of **3** in CH_2Cl_2 the yield of this oxidation was relatively poor, so we turned to sulfur oxidation of compound **1,** before coupling with **2**, which allowed to obtain the sulfoxide **4** in fairly good yield (Scheme 1). Coupling of **4** with the coumarin derivative **2** afforded **5** that was further reduced with PCl_3 and finally deprotected with TFA: CH_2Cl_2 (1:1 v:v) to yield **CAZ*** as the single Δ3 isomer.
To compare the activities of **CAZ** and **CAZ*** to those of their sulfoxides, we also prepared the corresponding sulfoxides **CAZ(S=O)** and **CAZ*(S=O)** by deprotection of the acids in **4** and **5**, respectively (Scheme S1).

1.1 Synthesis of 7-dimethylaminocoumarin-4-acetyl glycine (2)

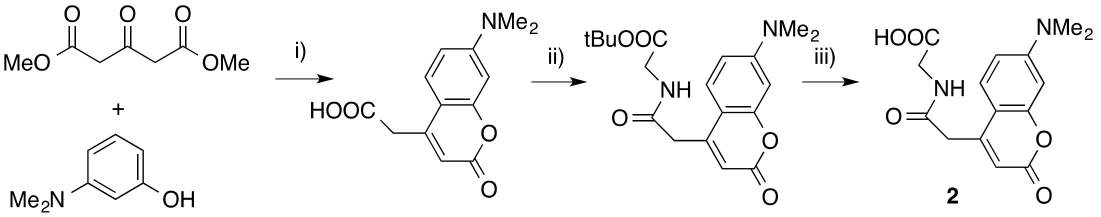

Scheme S2. Synthesis of 7-dimethylaminocoumarin-4-acetyl glycine

i) 1) ClTi(O*i*Pr)$_3$Toluene reflux 2) NaOH H_2O / MeOH, rt ii) Gly *t*Butyl ester EDC, HOBt, DIEA, DMF rt iii) TFA / CH_2Cl_2 0°C to rt

7-dimethylaminocoumarin-4-acetyl glycine (2) was synthesized in three steps from 3-dimethyl aminophenol and dimethyl 1,3-acetonedicarboxylate according previously described procedures, as shown in Scheme S2.

7-dimethylaminocoumarin-4-acetic acid.[5] The compound was obtained as a green-yellow powder in 40 % yield. ^{1}H NMR (250 MHz, DMSO-D6) δ (ppm): 12.60 (br, 1H), 7.46 (d, *J* = 9.0 Hz, 1H), 6.73 (dd, *J* = 9.0 & 2.6 Hz, 1H), 6.56 (d, *J* = 2.6 Hz, 1H), 6.04 (s, 1H), 3.77 (s, 2H), 3.02 (s, 6H). ESI$^+$ HRMS (*m/z*, CH_2Cl_2): calcd for $C_{13}H_{14}NO_4$ $[M+H]^+$ 248.0923; found: 248.0910.

7-dimethylaminocoumarin-4-glycine *tert*-butyl ester.[6] ^{1}H NMR (250 MHz, DMSO-D6), δ (ppm): 8.55 (t, *J* = 5.9 Hz, 1H), 7.57 (d, *J* = 9.0 Hz, 1H), 6.71 (dd, *J* = 9.0 & 2.6 Hz, 1H), 6.55 (d, *J* = 2.6 Hz,

1H), 6.05 (s, 1H), 3.75 (d, *J* = 5.9 Hz, 2H), 3.66 (s, 2H), 3.02 (s, 6H), 1.38 (s, 9H). ESI^+ HRMS (*m/z*, CH_2Cl_2): calcd for $C_{19}H_{25}N_2O_5$ $[M+H]^+$ 361.1763; found: 361.1749.

Compound 2. A final deprotection of 7-dimethylaminocoumarin-4-glycine *tert*-butyl ester (200 mg, 0.55 mmol) in a 1:1 mixture of CH_2Cl_2/TFA (4 mL) at r. t. overnight led to compound **2** in 92% yield. ^1H NMR (250 MHz, DMSO-D6) δ (ppm): 12.57 (br, 1H), 8.55 (t, *J* = 5.9 Hz, 1H), 7.57 (d, *J* = 9.1 Hz, 1H), 6.70 (dd, *J* = 9.1 & 2.5 Hz, 1H), 6.55 (d, *J* = 2.5 Hz, 1H), 6.06 (s, 1H), 3.78 (d, *J* = 5.9 Hz, 2H), 3.67 (s, 2H), 3.02 (s, 6H). APCI HRMS (*m/z*, CH_2Cl_2): calcd for $C_{15}H_{17}N_2O_5$ $[M+H]^+$ 305.1137; found: 305.1123

1.2. Synthesis of coumarin-labeled ceftazidime and sulfoxide derivatives

Diphenyldiazomethane was prepared as described by Davis *et al.*.[7] ^1H NMR (250 MHz, $CDCl_3$), δ ppm: 7.19 – 7.46 (m, 10H).

Compound 1. Synthesis of compound **1** was performed as previously described for cefotaxime by Zhang *et al*. .[8] Commercial ceftazidime (248 mg, 0.45 mmol) was dissolved in a 1:2 mixture of water and acetonitrile (3 mL), then *p*-toluene sulfonic acid (PTSA) (184 mg, 0.97 mmol) was added and the mixture was stirred for 10 min. An acetonitrile solution (3 mL) of diphenyldiazomethane (353 mg, 1.81 mmol) was added dropwise over 30 min. Upon consumption of Ph_2CN_2 the solution turned from pink to colorless. At the end of the reaction, when the acids were completely esterified, the solution remained pink. Then, the solvents were evaporated and the crude was diluted in CH_2Cl_2 (30 mL). After washing once with saturated aqueous NaCl solution, the organic layer was dried over Na_2SO_4 and concentrated, then precipitated into cyclohexane. Compound **1** was obtained exclusively in its Δ3 isomer. It's important to underline that neutralizing the reaction before workup is forbidden if we aim the exclusive formation of Δ3 isomer. Compound **1** was isolated as a white powder in quantitative yield upon precipitation of the crude from CH_2Cl_2 into cyclohexane. ^1H NMR (250 MHz, MeOD-D4) δ (ppm) (cationic part): 8.87 (d, *J* = 6.5 Hz, 2H), 8.49 (t, *J* = 8.1 Hz, 1H), 7.97 (dd, *J* = 8.1 & 6.5 Hz, 2H), 7.47 – 7.15 (m, 22H), 6.84 (s, 1H), 6.00 (d, *J* = 5.3 Hz, 1H), 5.58 (d, *J* = 14.6 Hz, 1H), 5.42 (d, *J* = 14.6 Hz, 1H), 5.25 (dd, *J* = 5.3 Hz, 1H), 3.73 (d, *J* = 18.7 Hz, 1H), 3.38 (d, *J* = 18.7 Hz, 1H), 1.63 (s, 3H), 1.62 (s, 3H); (anionic part) $PTSA^-$: 7.70 (d, *J* = 8.1 Hz, 2H), 7.21 (d, *J* = 8.1 Hz, 2H), 2.34 (s, 3H). ESI^+ HRMS (*m/z*, CH_2Cl_2): calcd for $C_{48}H_{43}N_6O_7S_2$ $[M]^+$ 879.2629; found: 879.2629.

Compound 4. To a CH_2Cl_2 solution (20 mL) of compound **1** (500 mg, 0.475 mmol) was added dropwise at 0 °C a CH_2Cl_2 solution (6 mL) of mCPBA (1.2 equiv, 98 mg, 0.57 mmol). The oxidation reaction proceeded instantaneously. Then, the solvent was evaporated, and the sulfoxide was isolated in 91% yield after precipitation in diethylether. ^1H NMR (500 MHz, DMSO-D6) δ (ppm) (cationic part): 8.90 (dd, *J* = 6.2 & 1.5 Hz, 2H), 8.63 (dd, *J* = 7.7 & 1.5 Hz, 2H), 8.15 (dd, *J* = 7.7 & 6.2 Hz, 2H), 7.52 – 7.50 (m, 2H), 7.47 – 7.44 (m, 2H), 7.41 – 7.36 (m, 6H), 7.35 – 7.30 (m, 6H), 7.29 – 7.21 (m, 6H), 6.94 (s, 1H), 6.80 (s, 1H), 6.74 (s, 1H), 6.23 (dd, *J* = 8.1 & 5.2 Hz, 1H), 5.61 (d, *J* = 14.9 Hz, 1H), 5.53 (d, *J* = 14.9 Hz, 1H), 5.15 (d, *J* = 5.2 Hz, 1H), 3.97 (d, *J* = 18.4 Hz, 1H), 3.61 (d, *J* = 18.4 Hz, 1H), 1.54 (s, 3H), 1.52 (s, 3H). (anionic part)) $PTSA^-$: 7.47 (d, *J* = 8.0 Hz, 2H), 7.11 (d, *J* = 8.0 Hz, 2H), 2.28 (s, 3H). ESI^+ HRMS (*m/z*, CH_2Cl_2): calcd for $C_{48}H_{43}N_6O_8S_2$ $[M]^+$ 895.2578; found 895.2526. El. anal. calcd. for $[C_{48}H_{43}N_6O_8S_2]^+(PTSA)^-$.0.6 PTSA.1.7$H_2O$: C, 59.20; H, 4.85; N, 7.00. Found: C, 58.98; H, 4.73; N, 6.75.

Compound 5. To a DMF solution of compound **4** (450 mg, 0.374 mmol on the basis of the el. anal.), compound **2** (173 mg, 0.413 mmol), HOAT (102 mg, 0.749 mmol) and HATU (284 mg, 0.749 mmol) was added N-methyl morpholine (124 μL, 1.13 mmol) and the reaction was stirred at r. t. overnight.

Precipitation by adding water (60 mL) gave a solid that was collected by filtration. Compound **5** was then purified over silica gel, eluted with CH_2Cl_2/MeOH (90:10, *v*:*v*) and isolated in 48% yield. ^{1}H NMR (500 MHz, DMSO-D6) δ (ppm) (cationic part): 12.61 (s, 1H), 8.98 (d, *J* = 8.1Hz, 1H), 8.90 (dd, *J* = 6.4 & 1.6 Hz, 2H), 8.63 (m, 2H), 8.15 (dd, *J* = 7.8 & 6.4 Hz, 2H), 7.58 (d, *J* = 9.1 Hz, 1H), 7.53 – 7.36 (m, 10H), 7.32 – 7.20 (m, 12H), 6.95 (s, 1H), 6.72 (dd, *J* = 9.1 & 2.6 Hz, 1H), 6.56 (d, *J* = 2.6 Hz, 1H), 6.26 (dd, *J* = 8.2 & 4.9 Hz, 1H), 6.07 (s, 1H), 5.60 (d, *J* = 15.3 Hz, 1H), 5.53 (d, *J* = 15.3 Hz, 1H), 5.16 (d, *J* = 4.9 Hz, 1H), 4.02 (d, *J* = 5.6 Hz, 2H), 3.98 (d, *J* = 18.5 Hz, 1H), 3.72 (s, 2H), 3.61 (d, *J* = 18.5 Hz, 1H), 3.02 (s, 6H), 1.57 (s, 3H), 1.55 (s, 3H). ESI$^+$ HRMS (*m/z*, CH_2Cl_2): calcd for $C_{63}H_{57}N_8O_{12}S_2$ $[M]^+$ 1181.3532; found 1181.3521. El. anal. calcd. for $[C_{63}H_{57}N_8O_{12}S_2]^+(PF_6)^-$ .1CH_2Cl_2.1H_2O: C, 53.75; H, 4.30; N, 7.83. Found: C, 53.45; H, 4.28; N, 7.69.

General procedure for deprotection in acid conditions: A solution (2.1 mL) containing CH_2Cl_2/TFA/TIS/H_2O (1:1:0.05:0.05 *v*:*v*:*v*:*v*) was added at 0 °C to protected compound **4** (67 mg, 0.056 mmol) or compound **5** (60 mg, 0.042 mmol). Then, the solution was stirred at r. t. for 2 h. After evaporating the solvents, the desired products were isolated in 95 % yield upon precipitation in diethylether.

Compound CAZ(S=O). ^{1}H NMR (500 MHz, DMSO-D6) δ (ppm): 8.98 (d, *J* = 6.9 Hz, 2H), 8.66 (t, *J* = 7.4 Hz, 1H), 8.59 (d, *J* = 8.2 Hz, 1H), 8.21 (dd, *J* = 7.4 & 6.9 Hz, 2H), 6.83 (s, 1H), 6.11 (dd, *J* = 8.2 & 5.0 Hz, 1H), 5.63 (d, *J* = 15.0 Hz, 1H), 5.55 (d, *J* = 15.0 Hz, 1H), 5.07 (d, *J* = 5.0 Hz, 1H), 3.88 (d, *J* = 18.1 Hz, 1H), 3.57 (d, *J* = 18.1 Hz, 1H), 1.47 (s, 3H), 1.46 (s, 3H), PTSA : 7.45 (d, *J* = 8.1 Hz, 2H), 7.11 (d, *J* = 8.1 Hz, 2H), 2.29 (s, 3H). ESI$^+$ HRMS (*m/z*, MeOH) calcd for $C_{22}H_{23}N_6O_8S_2$ $[M+H]^+$): 563.1013; found: 563.1023. El. anal. calcd. for $C_{22}H_{22}N_6O_8S_2$.1.5PTSA.1CF_3COOH.2H_2O: C, 42.68; H, 4.05; N, 8.66. Found: C, 42.53; H, 3.89; N, 8.71.

Compound CAZ*(S=O). ^{1}H NMR (500 MHz, DMSO-D6) δ (ppm): 12.66 (s, 1H), 8.98 (d, *J* = 6.8 Hz, 2H), 8.80 (d, *J* = 8.1 Hz, 1H), 8.66 (t, *J* = 7.6 Hz, 1H), 8.62 (t, *J* = 5.7 Hz, 1H), 8.21 (dd, *J* = 7.6 & 6.8 Hz, 2H), 7.57 (d, *J* = 9.0 Hz, 1H), 7.40 (s, 1H), 6.72 (dd, *J* = 9.0 & 2.6 Hz, 1H), 6.55 (d, *J* = 2.6 Hz, 1H), 6.13 (dd, *J* = 8.1 & 5.2 Hz, 1H), 6.07 (s, 1H), 5.63 (d, *J* = 14.7 Hz, 1H), 5.55 (d, *J* = 14.7 Hz, 1H), 5.08 (d, *J* = 5.2 Hz, 1H), 4.01 (d, *J* = 5.7 Hz, 2H), 3.89 (d, *J* = 18.1 Hz, 1H), 3.71 (s, 2H), 3.57 (d, *J* = 18.1 Hz, 1H), 3.02 (s, 6H), 1.48 (s, 3H), 1.47 (s, 3H). ESI$^+$ HRMS (*m/z*, MeOH) calcd for $C_{37}H_{37}N_8O_{12}S_2$ $[M+H]^+$ 849.1967; found: 849.2010. El. anal. calcd. for $C_{37}H_{37}N_8O_{12}S_2$.PF_6.CF_3COOH.3.5H_2O: C, 39.97; H, 3.87; N, 9.56. Found: C, 40.19; H, 3.61; N, 9.27.

Synthesis of CAZ*: To a DMF solution (5 mL) of compound **5** (243 mg, 0.17 mmol on the basis of the el.anal.) at -10 °C was added PCl_3 in three portions (2 equiv, then 1 and 1 equiv) (60 μL, 0.68 mmol), over 30 min. The disappearance of the sulfoxide can be monitored by mass spectrometry. After 50 min of reaction (reaction time less than 1 h to avoid product degradation), the crude solution was poored in a saturated NaCl solution (100 mL) leading to precipitation of the reduced product that was directly deprotected under the conditions described above. The product was purified over semipreparative HPLC: C18 column, Stability, 100 A°, 5 μm, 250x20 mm - injection volume 2 mL - mobile phase (A) water + 0.1% TFA and (B) acetonitrile + 0.1% TFA – elution gradient 100:0 to 0:100 over 40 min. ^{1}H NMR (500 MHz, DMSO-D6) δ ppm: 12.56 (s, 1H), 9.49 (d, *J* = 6.9 Hz, 2H), 8.60 (br, 1H), 8.57 (t, *J* = 7.5 Hz, 1H), 8.16 (dd, *J* = 7.5 & 6.9 Hz, 2H), 7.58 (d, *J* = 8.8 Hz, 1H), 7.30 (s, 1H), 6.72 (dd, *J* = 8.8 & 2.4 Hz, 1H), 6.54 (d, *J* = 2.4 Hz, 1H), 6.06 (s, 1H), 5.75 (dd, *J* = 8.1 & 5.1 Hz, 1H), 5.71 (d, *J* = 13.6 Hz, 1H), 5.07 (d, *J* = 13.6 Hz, 1H), 5.03 (d, *J* = 5.1 Hz, 1H), 4.01 (br, 2H), 3.71 (s, 2H), 3.48 (d, *J* = 17.8 Hz, 1H), 3.01 (s, 6H), 2.99 (d, *J* = 17.8 Hz, 1H), 1.40 (s, 3H), 1.35 (s, 3H). ESI$^+$ HRMS (*m/z*, MeOH): calcd for $C_{37}H_{37}N_8O_{11}S_2$ $[M]^+$ 833.2018; found: 833.2023.

2. Synthesis of ceftazidime labeled on the leaving group CAZ**

The titled compound was obtained by condensing (*Z*)-(*Z*)-2-(((1-(*tert*-butoxy)-2-methyl-1-oxopropan-2-yl)oxy)imino)-2-(2-(tritylamino)thiazol-4-yl)acetic methanesulfonic anhydride **8** with 1-(((6*R*,7*R*)-2,7-bis(((*tert*-butyl-λ^2-silyl)oxy)carbonyl)-8-oxo-5-thia-1-azabicyclo[4.2.0]oct-2-en-3-yl)methyl)-6-methoxyquinolin-1-ium **9** (Scheme S3). This acylation was accomplished as previously reported for the synthesis of other cephalosporin derivatives,[9] after activating **6** as mixed anhydride with $MeSO_2Cl$ and protecting both the acid and the amine of **7** as trimethylsilyl derivative. Final deprotection of **10** in pure TFA and HPLC purification afforded **CAZ**** as a mixture of syn/anti forms in a 70/30 ratio.

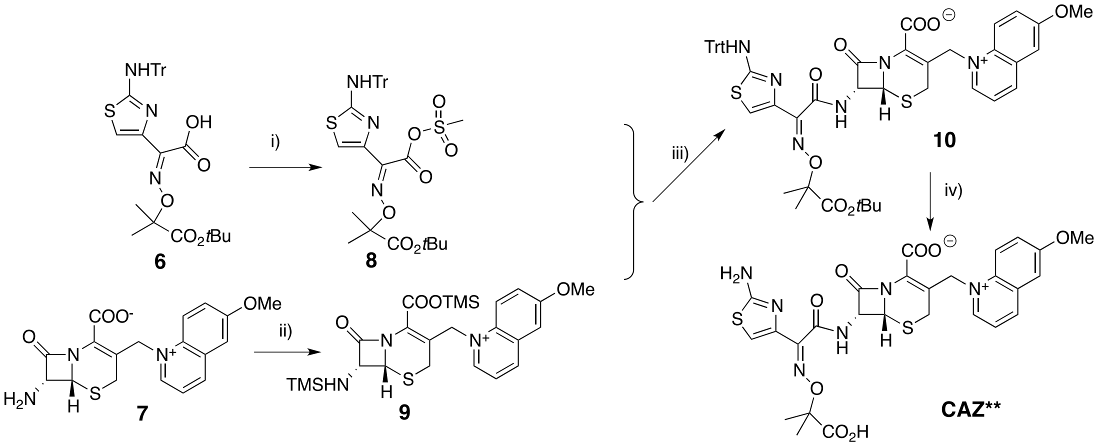

Scheme S3: Synthesis of **CAZ****

i) 1) CH_2Cl_2, 0°C 2) Et_3N (1.5 eq) -10°C 3) CH_3SO_2Cl (1.5 eq) ii) $[(CH_3)_3SiNH]_2CO$ (6 eq), CH_2Cl_2 r.t. iii) 1) CH_2Cl_2 0°C 2) Et_3N (1 eq), H_2O iv) CF_3COOH r.t.

Compound 6 was prepared as previously described in three steps from ethyl (*Z*)-2-(2-aminothiazol-4-yl)-2-(hydroxyimino)acetate after tritylation[10] and reaction with *tert*-butyl 2-bromo-2-methyl-propanoate followed by saponifaction.[11] ^1H NMR (250 MHz, $CDCl_3$) δ (ppm): 7.26 – 7.32 (m, 16H), 6.75 (s, 1H),1.56 (s, 6H), 1.42 (s, 9H). ESI^+ HRMS (*m/z*, CH_2Cl_2): calcd for $C_{32}H_{34}N_3O_5S$ $[M+H]^+$ 572.2214, found: 572.2202.

Compound 7 was obtained from (6*R*,7*S*)-3-(acetoxymethyl)-7-amino-8-oxo-5-thia-1-azabicyclo[4.2.0]oct-2-ene-2-carboxylic acid by preparing the iodomethyl intermediate with NaI in CH_3CN and silylating the acid with bistrimethylsilylacetamide according to a procedure previously described.[12] ^1H NMR (500 MHz,CF_3COOD) δ (ppm): 9.37 (d, *J* = 5.8 Hz, 1H), 9.35 (d, *J* = 8.3 Hz, 1H), 8.71 (d, *J* = 9.7 Hz, 1H), 8.35 (dd, *J* = 8.3 & 5.8 Hz, 1H), 8.31 (dd, *J* = 9.7 & 2.6 Hz, 1H), 7.98 (d, *J* = 2.6 Hz, 1H), 6.86 (d, *J* = 16.4 Hz, 1H), 6.46 (d, *J* = 16.4 Hz, 1H), 5.90 (d, *J* = 5.0 Hz, 1H), 5.76 (d, *J* = 5.0 Hz, 1H), 4.35 (d, *J* = 18.1 Hz, 1H), 4.16 (d, *J* = 18.1 Hz, 1H), 3.99 (s, 3H). ESI^+ HRMS (*m/z*, CH_3CN): calcd for $C_{18}H_{18}N_3O_4S$ $[M+H]^+$ 372.1013; found: 372.1002.

Compound 10 was prepared in three steps following a procedure previously described.[9]

*Preparation of **8***: a solution of compound **6** (84.6 mg, 0.27 mmol) in CH_2Cl_2 (2 mL) was cooled to 0°C, then Et_3N (0.40 mmol, 1.5 eq) was added. After cooling the solution to -10°C, $MeSO_2Cl$ (0.48 mmol) was added and the solution was stirred for 30 min.

*Preparation of **9***: bis(trimethylsilyl)urea (1.62 mmol, 6 eq) was added at r.t. to a suspension of **7** (100 mg, 0.27 mmol) in CH_2Cl_2 (2 mL) and stirred for 2.5 h.

Acylation reaction: The solution of the mixed anhydrid **8** was rapidly added to the solution of **9** cooled to 0°C and the mixture was stirred in an ice bath for 45 min. Then water (8 mL) was added and the organic layer was extracted, dried over Na_2SO_4 and evaporated to dryness. Finally, the crude was triturated with Et_2O to get **10** as a solid that was dried and used in the next step without further purification.

Deprotection of 10 and isolation of CAZ**: this step was performed by stirring at 0°C a solution of **10** in pure CF_3COOH (850 µL). After evaporating the solvent, addition of diethyl ether promoted precipitation of **CAZ**** that was isolated and further purified over semi-preparative HPLC : Stability C18 column, 100 A°, 5 µm, 250x20 mm - injection volume 2 mL - mobile phase : (A) water + 0.1% TFA, (B) CH_3CN + 0.1% TFA - elution gradient A/B from 100:0 to 60:40 over 20 min. The product was isolated in 50% yield (85 mg). ^{1}H NMR (500 MHz, CF_3COOD): ratio Syn/Anti 70/30; *Syn isomer* δ (ppm): 9.46 (d, J = 7.1 Hz, 2H), 8.83 (d, J = 9.7 Hz, 1H), 8.47 (t, J = 9.7 Hz, 1H), 8.43 (dd, J = 9.7 & 2.7 Hz, 1H), 8.11 (d, J = 2.7 Hz, 1H), 7.84 (s, 1H), 6.83 (d, J = 16.1 Hz, 1H), 6.59 (d, J = 4.9 Hz, 1H), 6.51 (d, J = 16.1 Hz, 1H), 5.87 (d, J = 4.9 Hz, 1H), 4.57 (s, 3H), 4.13 (d, J = 18.7 Hz, 1H), 3.83 (d, J = 18.7 Hz, 1H), 2.23 (s, 3H), 2.21 (s, 3H). *Anti isomer* δ (ppm): 9.46 (d, J = 7.1 Hz, 2H), 8.83 (d, J = 9.7 Hz, 1H), 8.47 (t, J = 7.1 Hz, 1H), 8.43 (dd, J = 9.7 & 2.7 Hz, 1H), 8.11 (d, J = 2.7 Hz, 1H), 7.90 (s, 1H), 6.67 (d, J = 5.1 Hz, 1H), 5.83 (d, J = 5.1 Hz, 1H), 5.65 (d, J = 17.1 Hz, 1H), 5.58 (d, J = 17.1 Hz, 1H), 4.54 (s, 3H), 4.42 (d, J = 18.7 Hz, 1H), 4.18 (d, J = 18.7 Hz, 1H), 2.28 (s, 3H), 2.26 (s, 3H). ESI$^+$ HRMS (*m/z*, MeOH): calcd. for $C_{27}H_{27}N_6O_8S_2$ $[M + H]^+$ 627.1326; found: 627.1337.

Abbreviations

HOAt, 1-hydroxy-7-azabenzotriazole; HATU, 1-[Bis(dimethylamino)methylene]-1*H*-1,2,3-triazolo[4,5-b]pyridinium-3-oxid hexafluorophosphate; HOBt, *N*-hydroxybenzotriazole; NMM, *N*-methylmorpholine; EDC, *N*-Ethyl-*N′*-(3-dimethylaminopropyl)carbodiimide ; DIEA, *N,N*-diisopropylethylamine; TIS, triisopropylsilane; PTSA, *p*-toluenesulfonic acid; PTSA$^-$, *p*-toluenesulfonate; TFA, trifluoroacetic acid.

References

1. Cheng, Y. *et al. Angew. Chem. Int. Ed.* **53**, 9360-9364 (2014).
2. Xiao, J.-M. *et al. Eur. J. Med. Chem.* **59**, 150-159 (2013).
3. Farina, V. *et al. J. Org. Chem.* **54**, 4962-4966 (1989).
4. Kaiser, G.V. *et al. J. Org. Chem.* **35**, 2430-2433 (1970).
5. Wirtz, L., Kazmaier, U. *Eur. J. Org. Chem.* **35**, 7062-7065 (2011).
6. Alexander, M. D. *et al. ChemBioChem.* **7**, 409-416 (2006).
7. Davis, P.J. *et al. Tetrahedron Lett.* 52, **2011**, *52*, 1553 (2011).
8. Zhang, J. *et al. Angew. Chem. Int. Ed.* **51**, 1865-1868 (2012).
9. Wirth, D.D, *Tetrahedron* **49**, 1535-1540 (1993).
10. Yamamoto, H. *et al. Bioorg. Med. Chem.* **10**, 1535-1545 (2002).
11. Heymes, R. *Can. Patent* CA 1132575 A2 19820928 (1982).
12. Lattrell. R. *et al. J. Antibiotics* 1874 (1988).

3. ^{1}H NMR Spectra

CAZ(S=O), NMR, 500MHz, DMSO-d6

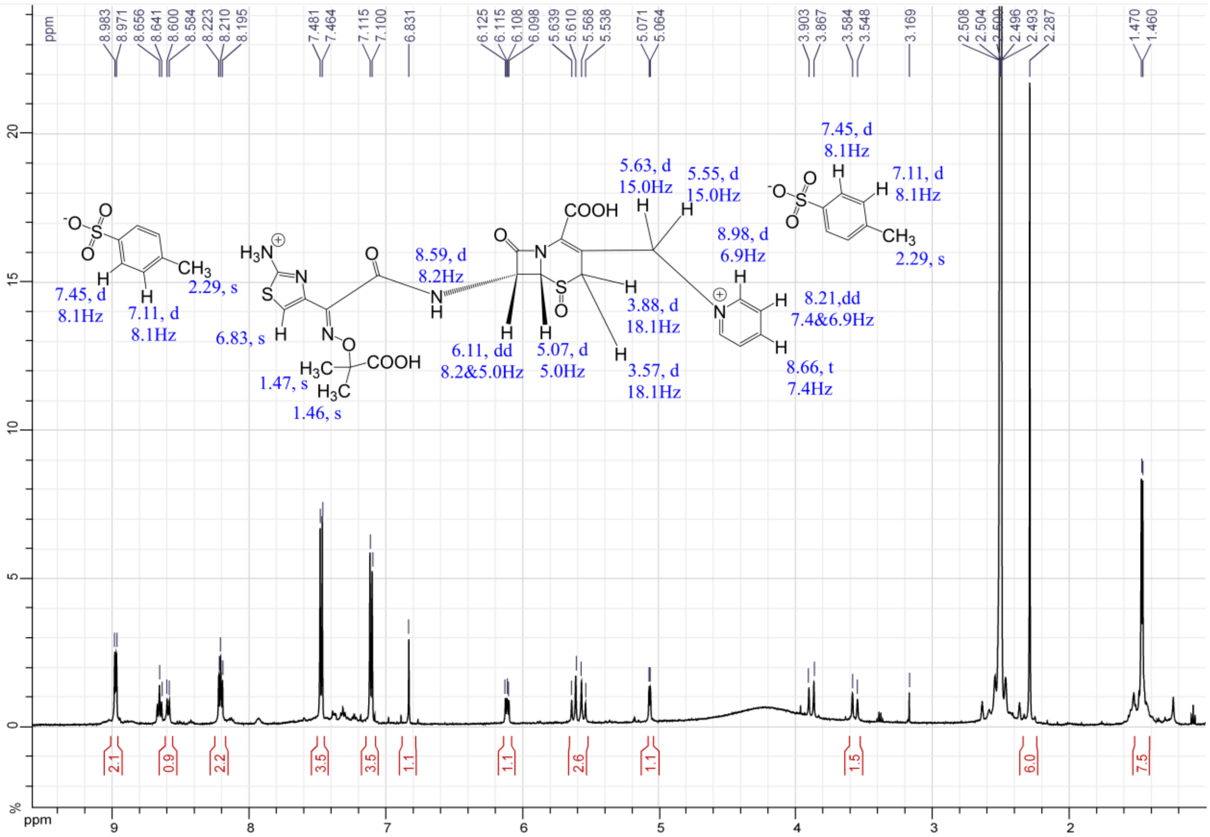

CAZ*(SO), NMR, 1H, 500MHz, DMSO-d6

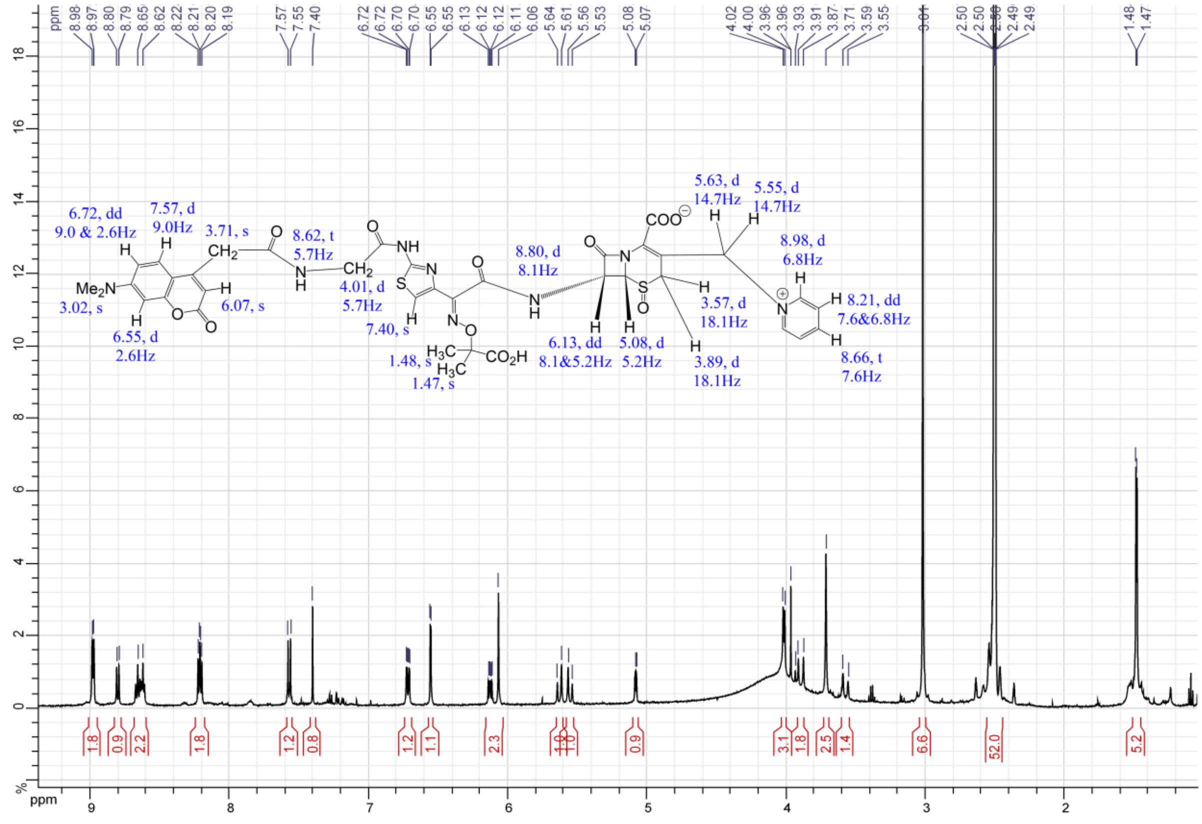

CAZ*, NMR, 1H, 500MHz, DMSO-d6

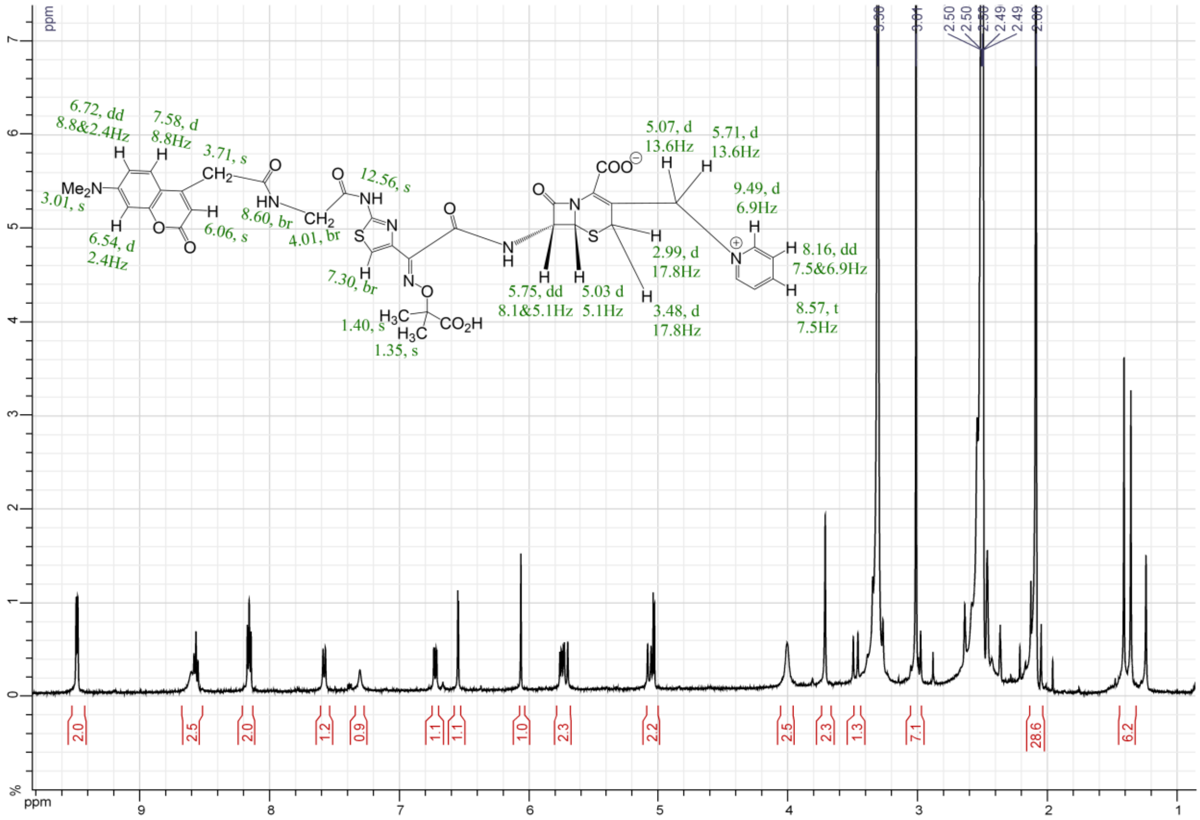

CAZ**, NMR 500MHz, d-TFA

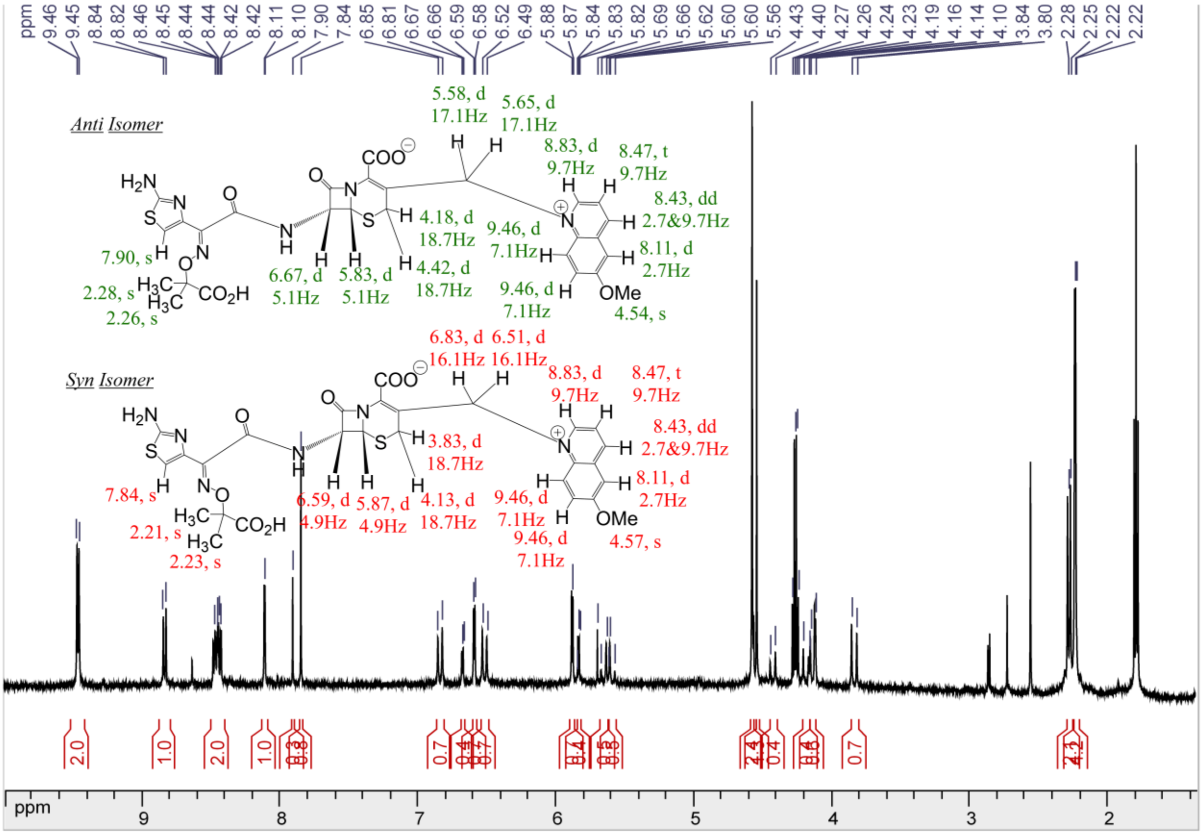

Supplement: Supplementary file 1 — SUPPLEMENTARY INFORMATION [file 41598_2017_945_MOESM1_ESM.pdf]
